# Supplementary material for: The source of the Black Death in fourteenth-century central Eurasia
Source: Nature. 2022 Jun 15;606(7915):718–24. doi: 10.1038/s41586-022-04800-3 (PMC9217749; doi:10.1038/s41586-022-04800-3)
Supplement: Supplementary file 1 — Supplementary Sections 1–4 and References. [file 41586_2022_4800_MOESM1_ESM.pdf]

nature portfolio

https://doi.org/10.1038/s41586-022-04800-3

Supplementary information

The source of the Black Death in fourteenth-century central Eurasia

In the format provided by the authors and unedited

Supplementary Information

The source of the Black Death in fourteenth-century central Eurasia

Maria A. Spyrou*, Lyazzat Musralina, Guido A. Gnecchi Ruscone, Arthur Kocher, Pier-Giorgio Borbone, Valeri I. Khartanovich, Alexandra Buzhilova, Leyla Djansugurova, Kirsten I. Bos, Denise Kühnert, Wolfgang Haak, Philip Slavin*, Johannes Krause*

*To whom correspondence should be addressed: maria.spyrou@ifu.uni-tuebingen.de; philip.slavin@stir.ac.uk; krause@eva.mpg.de

Table of Contents

Supplementary Information 1 3
Second Plague Pandemic origin theories 3
The East Asian hypothesis 3
The central Eurasian hypothesis 4
The greater Pontic-Caspian basin hypothesis 4
Alternative hypotheses 5
Supplementary Information 2 6
Archaeological and historical information 6
Study background 6
Historical context of the 14th-century Chüy Valley region 6
List of institution archives that were examined for this study 8
The Kara-Djigach cemetery 9
Background information on the cemetery and 19th century excavations 9
Supplementary Fig. 1 11
Tombstone dataset from Kara-Djigach 12
Supplementary Fig. 2 14
Information on individuals from Kara-Djigach analysed in this study 15
Supplementary Fig. 3 15
The Burana cemetery 19
Background information on the cemetery and 19th century excavations 19
Tombstone dataset from Burana 20
Information on the individuals from Burana analysed in this study 21
Supplementary Fig. 4 21
Toponymic indications of mobility in Kara-Djigach and Burana 22
Supplementary Information 3 25
Human aDNA analysis 25
Methods 25
Human aDNA data processing 25
Genotyping and PCA 25
Genetic relatedness 26
Ancestry modelling 26
Results 27
Human genomic results from Kara-Djigach and Burana 27
Supplementary Fig. 5 28
Supplementary Information 4 29
Additional supplementary figures 29
Supplementary Fig. 6 29
Supplementary Fig. 7 30
Supplementary Fig. 8 31
Supplementary Fig. 9 32
Supplementary Fig. 10 33
Supplementary Fig. 11 34
Supplementary Fig. 12 35
Supplementary Fig. 13 36
Supplementary table descriptions 37
Supplementary References 39

Supplementary Information 1

Second Plague Pandemic origin theories

The exact location and timing of the event(s) that triggered the 'Second Plague Pandemic' are topics that have been a major focus of research and still remain unresolved. Although discussions relating to these topics are ongoing for more than two centuries, hypotheses on the pandemic's initial source currently support a number of possible locations ranging from the Pontic-Caspian steppe to eastern Asia. Roughly speaking, it is possible to extrapolate four different origins hypotheses: (1) the East Asian or 'Chinese' hypothesis; (2) the central Eurasian hypothesis, (3) the greater Pontic-Caspian basin hypothesis, and (4) alternative hypotheses. Below we provide a comprehensive, but likely not exhaustive, overview of all four categories.

The East Asian hypothesis

The hypothesis of an origin within eastern Asia goes back to at least Joseph de Guignes' *Histoire générale des Huns, des Turcs, des Mongols et des autres Tartares occidentaux* (1756-8)[1]. De Guignes (1721-1800) was a French orientalist and sinologist, with a particular interest in the history of Turkic peoples. In advancing the Chinese origin theory, he referred to a Mamluk source which had stated that the plague began in the 'lands of the Great Khan', and to Chinese annals depicting natural disasters and epidemics in the 1330s and 1340s. De Guignes's views have been taken up by Kurt Sprengel in his *Beiträge zur Geschichte der Medicin* (1794)[2]. Sprengel was followed by Friedrich Schnurrer's *Chronik der Seuchen in Verbindung mit den gleichzeitigen Vorgängen* (1823)[3], and Justus Hecker's 1832 *Der schwarze Tod im vierzehnten Jahrhundert*[4]. According to both Schnurrer and Hecker, the Black Death commenced in China, before spreading through Asia into Crimea. Hecker's work was subsequently translated into English (1833), and it was one of the sources used by Francis Aidan Gasquet in his 1893 *Great Pestilence (A.D. 1348-9)*, where he postulated, in a similar fashion to Schnurrer and Hecker, that it was Italian merchants that brought the plague from China to Crimea, via Asia, in trading caravans[5]. Similarly, Johannes Nohl stated in his 1924 *Der schwarze Tod: eine Chronik der Pest 1348-1720* that the plague started in China and spread into Europe along trade-routes, via India, Persia and Russia[6]. The East Asian origins hypothesis has been advanced ever since. While some historians and microbiologists speak of 'China'[7-10], or 'East Asia'[11], or even the 'Far East'[12,13] in general terms, some scholars pinpoint specific regions overlapping with the borders of Late Imperial and Republican China. Based on modern *Y. pestis* genomic data, Cui et al. identified a star-like divergence of four lineages (commonly designated as Branches 1, 2, 3, 4) that closely preceded the Black Death to have occurred sometime between the 12th and 14th centuries AD[14]. Moreover, they suggested that *Y. pestis* likely originated in the Tibetan-Qinghai Plateau, from where it spread on multiple occasions. Building on this hypothesis, Robert Hymes suggested that from the Tibetan-Qinghai Plateau, plague may have spread to the rest of China through the Gansu corridor, in conjunction with early Mongol campaigns of the 1210s-1230s[15]. Likewise, Bruce Campbell accepted the later thirteenth-century Tibetan-Qinghai Plateau as the birthplace of the Second Pandemic, but held that the spill-over from

sylvatic rodents to humans occurred around Issyk-Kul in the 1330s[16]. Conversely, William McNeill conceded the Yunnan-Burma border in the eastern edge of the Himalayas, as a possible candidate for the geographic origins of the Second Pandemic[17].

The central Eurasian hypothesis

The central Eurasian hypothesis originated with the 1890 publication of the second instalment of Daniel Chwolson's textual edition of tombstone inscriptions from the Kara-Djigach and Burana cemeteries. Chwolson noted the excessive number of tombstones from Kara-Djigach dated to a single year (1338-39), and that some stated that individuals "died of pestilence" (*mīt[ā] [bi-]mawtānā* in Syriac)[18]. Chwolson reiterated this connection in the third part of his publication (1897)[19] and was the first to speculate that the 1338-9 epidemic was associated with the Black Death - albeit without hypothesising whether the plague started around Kara-Djigach, or further east.

The first scholar to make the Kara-Djigach inscriptions and their possible connection to the Black Death known to a wider readership was John Stewart, in his 1928 monograph on medieval East Syriac church[11]. Although Stewart hypothesised that the fourteenth-century plague commenced in 'Eastern Asia', he was preceded by some five years by Wu Lien-teh (1879-1960), who was the first to argue clearly for the central Eurasian origins of the Black Death – without mentioning any connection with the Kara-Djigach tombstones[20,21]. It was Georgii Kalina, a microbiologist studying plague foci across central Eurasia, who, in his 1936 monograph, was the first to postulate that the Black Death might have originated in one of Tian Shan reservoirs around Kara-Djigach[22]. Unlike Wu Lien-teh, he was aware of the Kara-Djigach inscriptions.

The Chüy Valley tombstones and Wu Lien-teh's theory of central Eurasian origins of the fourteenth century plague, became more widely known due to a comprehensive study by R. Pollitzer, a World Health Organisation plague ecologist[23], who had himself collaborated with Wu Lien-teh since the 1930s. Ever since, the central Eurasian origins theory was adopted by a number of historians, and it became one of the leading theories on the pandemic's origins. Just as with the East Asian hypothesis, some adherents of the central Eurasian hypothesis referred to this wider geographic region without further specification[24-31], while some historians spoke about the 'Central Asian steppe'[32,33], and others suggested that the Black Death commenced in/around the Tian Shan region[34-37]. Most recently, a research article by Monica Green put forward a new hypothesis through a synthesis of historical, genetic and ecological data, in which the pandemic's emergence was placed in central Eurasia, possibly around the Tian Shan region, in the early thirteenth-century[38]. It was further suggested that as a result of this emergence, new plague lineages spread across Eurasia in the course of the 13th century via military campaigns of the expanding Mongol Empire and that the reservoir that radiated the Black Death was seeded around the Caucasus-Volga region in the 1250s[39,40].

The greater Pontic-Caspian basin hypothesis

The Pontic-Caspian basin hypothesis encompasses regions of the Pontic-Caspian steppe, the Volga region, the Caucasus, eastern Anatolia, northern Iraq and northern Iran. Its history goes

back to at least Charles Creighton's 1891 *History of Epidemics in Britain*, where he conceded that the plague may have existed in a latent form in the vicinity of Caffa in Crimea, and from there it would later spread eastwards to China[41]. Fourteen years later, Creighton's theory was cited by W.J. Simpson who, without rejecting it, stated that establishing the geographic origins of the Black Death may never be possible[42]. The hypothesis that the Black Death may have originated in foci of the Pontic-Caspian steppe was reiterated in the late 1950s and 1960s by plague biologists[43,44]. Building upon their work, but with a slightly diverted geographic focus, J. Norris, suggested that there is no evidence to connect 'pestilence tombstones' from the Chüy Valley with the either Black Death in particular or with 'plague' in general and that the fourteenth-century plague originated in a reservoir within eastern Anatolia/northern Iraq, and not in central Eurasia[45]. Similarly, Ole Benedictow also adopted the proximate origins hypothesis within the Pontic-Caspian region[46-49]. John Alexander expanded the geographic range of the possible foci in which the Black Death was thought to have originated, in a territory stretching across the Pontic-Caspian steppe, the Caucasus and into northern Iran[50]. Similarly, Suponitskii and Suponitskaya spoke about the 'Great Eurasian plague fracture', stretching from the north Caspian shore to eastern Kurdistan and consisting of 'relict' plague foci[51]. As noted above, the Caucasus-Volga region was also suggested by M. Green as a possible proximate origin of the Black Death (in contrast to its ultimate origins in central Eurasian)[38,40]. Most recently, the Armenian Highlands around the Lake Van region in eastern Anatolia have been suggested by T. Khaydarov as the proximate source of the Black Death[52].

Alternative hypotheses

Over the last years, a number of alternative hypotheses on the Black Death's origins have been put forward. A number of scholars, since August Hirsch (1859)[53], have suggested and origin within India[54,55], with one author specifying the Garhwal and Kumaon regions of southern Himalayas, on the basis of Ibn-Battuta's reference to an epidemic there[56]. Moreover, in a recent aDNA publication, Namouchi and colleagues advanced the idea that the plague may have spread from the western Urals, which was misidentified as the 'Land of Darkness' of medieval Islamicate geographers[57]. Subsequently, P. Slavin suggested a possible origin either in western Siberia or the Tian Shan region, and referred to western Siberia as the true designation for the 'Land of Darkness'[37]. Finally, the Mongolian-Manchurian steppe/Gobi Desert has also been mentioned as another possible source by W. McNeill, R. S. Gottfried and J. Kelly[17,35,58].

Supplementary Information 2

Archaeological and historical information

Study background

The Black Death is known to have reached western Eurasia by AD 1345/1346, from where it disseminated west of the Black Sea in 1347[46,47,59]. However, concrete information on its earlier history is currently lacking. To date, none of the existing hypotheses on the pandemic's origins have been sufficiently addressed due to the limited number of published archaeological sites from the proposed areas, the scarcity of historical investigations focusing on events closely preceding the Black Death and the absence of ancient genomic data from regions outside of Europe. While a number of associated archaeological excavations beyond our knowledge may be ongoing in any of those candidate regions, at present, the most widely discussed archaeological evidence of a 14th-century mortality crisis beyond western Eurasia, which was contemporaneous, or closely preceded, the initiation of the Black Death, derives from Christian cemeteries situated in the Chüy Valley, west of the Lake Issyk-Kul in modern-day northern Kyrgyzstan[37].

A possible association between these sites and the Black Death has been advanced since the 1890 publication by D. Chwolson[18]. At present, all pestilence-stating inscriptions (ten in total[37]) have been identified within one of the two discussed sites, the Kara-Djigach cemetery. Although the Syriac term *mawtānā* provides an explicit description of an infectious disease-associated cause of death for the buried individuals, any specific association of this event with the bacterium *Y. pestis* and, more specifically, with the Black Death has not been so far demonstrated and is forming the basis and motivation for the present study. In particular, specimens from the Kara-Djigach and Burana cemeteries, which were excavated in the late 19th century by N. Pantusov and A. Fetisov, comprise the aDNA dataset analysed in this study and are discussed in detail within the sections below.

Historical context of the 14th-century Chüy Valley region

The Chüy Valley, alongside other parts of the Semirech'ye (Zhetysu) region, fell under the Mongol control in the course of the AD 1218-9 campaign against the Qara-Khitai Empire. The imposition of the Mongol control in the next two decades or so witnessed the forced transformation of local arable and urban spaces into grazing grounds for Mongol herds. Despite much ecological, and socio-economic damage, a revival came in the late 1240s-1250s, with old communities recovering and new settlements emerging[37].

The Kara-Djigach cemetery was attached to one such new community. The community's residents belonged to the so-called 'Church of the East' (also known as the 'East Syriac' or 'Nestorian' Church), accused by Orthodox and Catholic authorities of not accepting the divine nature of Jesus Christ. By contrast, Mongol authorities of the Chaghadaid Khanate (overlapping, roughly speaking, with central Eurasia), as Qara-Khitai before them, were pronouncedly tolerant of the religious dogmas and practices of local conquered populations,

such as Christian, Muslim, Buddhist or Shamanist groups, certainly until their conversion to Islam from the 1320s-1330s onwards[60,61].

The available textual, archaeological, and epigraphic evidence suggests that the two settlements had somewhat different histories. The site of Burana seems to have been associated with the city of Balasaghun, founded presumably by the Sogdians in the tenth century. The city grew into a prominent cultural and commercial centre in central Eurasia, and later became the capital of the Qarakhanid and Qara-Khitai empires. The city is considered to have been destroyed during the Mongol conquests, but evidently came back to life shortly after, as indicated in coins minted in a local mint into the 1230s[62,63]. Because the vast majority of tombstones identified in Burana are undated, it is impossible to establish an approximate beginning of the East Syriac community there. It may have existed there before the Mongol attack, but it may have equally settled there in its aftermath, with immigrants arriving from places like Almaliq and Kashgar, seeking to profit from favourable conditions along the long-distance trans-Asian trade routes, and the prominent position that Burana was playing in this context – if the association of the Burana site with Balasaghun is indeed accurate. At present, the earliest dated tombstone from this site is from AD 1266-7[64].

By contrast, there is no evidence – textual or archaeological – that the Kara-Djigach settlement existed in pre-Mongol times. As it is hinted in some tombstones designating some individuals as 'of Almaliq' (*Almāligayā*/*Almāligaytā*) and 'of Kashgar' (*Kaškarayā*) (two prominent cities in western Xinjiang)[18,19], its Christian community seems to have been founded by immigrants from other parts of central Eurasia, at some point before AD 1248 (the date of the earliest identified headstone), possibly fleeing from widespread destruction inflicted by the Mongols in the 1220s-1230s, or – to the contrary – attracted by new commercial prospects encouraged by the Mongol rule of central Eurasia, from the 1240s onwards. Indeed, Kara-Djigach, just as Burana and other Semirech'ye settlements, was conveniently located at the centre of the northern branch of these trans-Asian trade routes, most likely functioning as a trade-and-rest station for merchants and their caravans[16,37].

The involvement of Kara-Djigach and Burana residents in commercial activities (local, regional, and long-distance) is revealed by both textual and archaeological evidence. European travellers, such as William Rubruck (travelling in 1253)[65] and Marco Polo (travelling in 1271-5)[66], commented on the financial activities of central Eurasian Christian communities. Among excavated grave goods, we find pearls (harvested in far waters - the Persian Gulf, as well as the Pacific and Indian oceans), corals and *Marginella* shells (harvested, most likely, in the Mediterranean Sea), as well as both precious and non-precious metal objects, silk and golden brocade cloths (see description below in the section *"The Kara-Djigach cemetery"*). Moreover, numismatic evidence from Kara-Djigach and Burana sheds some additional light on the local community's involvement in long-distance trade with regions as far as Khwarazm, Transoxiana, northern Afghanistan, northern Iran, and the Volga basin (see description in the section *"Kara-Djigach numismatic evidence"*). Balasaghun certainly had its own mint (most likely discontinued after the 1230s), and it has been suggested that Kara-Djigach may have had

its own mint, producing low-quality imitation coins, with a very limited geographic circulation within the Chüy Valley of central Eurasia[67].

List of institution archives that were examined for this study

The following institutions archives were examined for identifying and translating information associated with the excavations and archaeological surveys of the Kara-Djigach and Burana cemeteries that took place in the years 1885, 1886 and 1892:

- *IAC =The Imperial Archaeological Commission* (Imperatorskaya Arkheologicheskaya Komissiiya). A state-sponsored archaeological society based in St. Petersburg, operating between 1859 and 1919, after which it was taken over by the Institute of Material Culture History of the Russian Academy of Sciences
- *Kunstkamera* = The Kunstkamera museum, St. Petersburg, (also known as, the Peter the Great Museum of Anthropology and Ethnography)
- *Hermitage* = The State Hermitage Museum, St. Petersburg
- *LSU, FA* = Leningrad State University, Faculty of Anthropology (from 1724 to 1914: St Petersburg University; from 1914 to 1924: Petrograd University; from 1924 to 1991: Leningrad University; from 1991-present: St Petersburg State University)
- *AIIMKRAN* = The Archive of the Institute of Material Culture History of the Russian Academy of Sciences, St. Petersburg (Arkhiv Instituta Istorii Material'noi Kul'tury Rossiiskoi Akademii Nauk)

The Kara-Djigach cemetery

Individuals analysed:

- BSK001 (176/4; Grave 19/20)
- BSK002 (176/5; Grave 22)
- BSK003 (176/7; Grave 28/29)
- BSK006 (5559/1; Grave 6)
- BSK007 (5559/2; Grave 9)

Background information on the cemetery and 19th century excavations

The location of the Kara-Djigach Christian cemetery (coordinates: N42°48'21.68" E74°41'8.77") can be established at ~11 km to the south-east of Bishkek (at the site of the Kara-Djigach village). To the south is the Ala-Too Range, forming a part of the northern Tian Shan. The cemetery was situated between two irrigation canals (*aryks*), Djalair in the west, and Aksak-Büry in the east. The total area of the cemetery was reported to have been about three *desyatinas* (8.1 acres=32,780 square metres, namely, about 256x128m) (see Fig. 1b in the main text).

The cemetery was first discovered in June 1885 by a local land surveyor, V. A. Andreyev (*AIIMKRAN, Fund 1, Inventory 1, File 40b, fol. 62r*). The first excavation, which was initiated by N. N. Pantusov and directed by A. A. Fetisov, took place between October and early November 1885, in the course of which two graves (containing four individuals) were excavated at the south-western end of the cemetery and 35 tombstones (mostly in Syriac, but some in Turkic) were collected. Of these, three tombstones were sent to the Imperial Archaeological Commission (*IAC*) in St. Petersburg[18] (*AIIMKRAN, Fund 1, Inventory 1, File 40b, fol.42v*), a further three were sent to the *West-Siberian Division of the Imperial Russian Geographic Society* in Omsk, two were given to Neophyt, Russian Orthodox Bishop of Tashkent and Turkestan and an additional stone was sent to the *Kazan Society of History, Archaeology and Ethnography*[68]. In addition, a further two tombstones were sent in early 1886 to the First Siberian University of Tomsk (founded in 1878)[69] and were subsequently handed over to the Geological Collection of the Novosibirsk District Museum shortly after the end of World War II[70]. The remaining 24 were sent to N. Pantusov's headquarters in Verny (modern-day Almaty). At a later stage (exact time unknown), one of the tombstones was brought to Alnwick Castle, in Northumberland, England, most likely through the efforts of Henry Percy, Earl Percy (1871-1909), son of Henry Percy, 7th Duke of Northumberland[71]. In 1950, the stone was purchased by the Gulbenkian Museum, University of Durham[71]. An additional tombstone excavated during the same season, was acquired, by Osborne Beauclerk, 12th Duke of St Albans (1874-1964), who donated it in 1905 to the British Museum (museum number: 1905,0320.1; https://www.britishmuseum.org/collection/object/H_1905-0320-1). Overall, during the 1885 fieldwork season, a total of 611 tombstones were counted by A. Fetisov, which

were estimated to have been about one-fifth of the total ~3,000 burials present at that site[68] (*AIIMKRAN, Fund 1, Inventory 1, File 40b, fols.42v-43r*).

During the 1886 excavations (August 1886), supervised by N. Pantusov and A. Fetisov[68], a total of 83 graves with 90 skeletons were excavated at four main areas of the Kara-Djigach cemetery (*AIIMKRAN, Fund 1, Inventory 1, File 40b, fols.2r-31r*). The majority of the excavated burials were at the south-western end of the cemetery (56 in total, nos. 1-38, 60-77), specifically to the south and south-east of a structure that N. Pantusov referred to as "Chapel 1" (see Fig. 1b of main text). Another area of 14 excavated graves (numbers 44-55, 58-9) was located in the north-western corner of the cemetery, to the south west of a structure named "Chapel 2". Within Chapel 2, three graves (graves A-C) were excavated. At the southern corner of the cemetery, another area, containing two graves (56 and 57) was excavated. In addition, eight graves were excavated on three hills. These were 'Hill A' at the centre of the cemetery that contained graves 39-42, Hill B at the centre-east containing one un-numbered grave and Hill C at the north-east that contained graves 43a-43c.
From the total of 83 graves containing 90 skeletons, 29 skulls were retrieved and dispatched to Verny, modern-day Almaty (Graves 1-9, 13, 16, 20-24, 26, 28-29, 32, 35-7, 43b, 44, 46-47, 59, 68, 75). Of these, 27 (all, except skulls from Graves 8 and 44) were sent to St. Petersburg in April 1888, to the custody of the *IAC*, which moved them shortly after to the *Kunstkamera*. Our analyses of the excavation reports (*AIIMKRAN, Fund 1, Inventory 1, File 40b, fols. 85v, fols. 118r-120r;* the catalogue by Y. Ludevig, 1904[72]; and the *Kunstkamera craniological collection: files 176 and 5551*) as well as the publications by D. Chwolson[18] and S. Slutskii[73], show that of the 27 skull specimens sent to the *Kunstkamera* seven are undated or were found in burials with no associated tombstones, four are, respectively, from the years 1300, 1327, 1330 and 1336, and 16 are from the epidemic years AD 1338-1339. At present, 18 skull specimens from Kara-Djigach remain at the *Kunstkamera*[74] (*AIIMKRAN, Fund 1, Inventory 1, File 40b, fols. 85v, fols. 118r-120r; Kunstkamera craniological collection: files 176 and 5551*).

Importantly, the association between grave numbers, tombstones and the identity of buried individuals can be established as, in the course of the summer 1886 excavations at Kara-Djigach, all tombstones were marked with the same number as their associated graves[75]. Likewise, excavated human remains were assigned the same number as their associated graves and tombstones. Moreover, in his subsequent work, D. Chwolson maintained all original grave/tombstone numbers assigned by N. Pantusov during the August 1886 excavations next to his own editorial numbers[18].

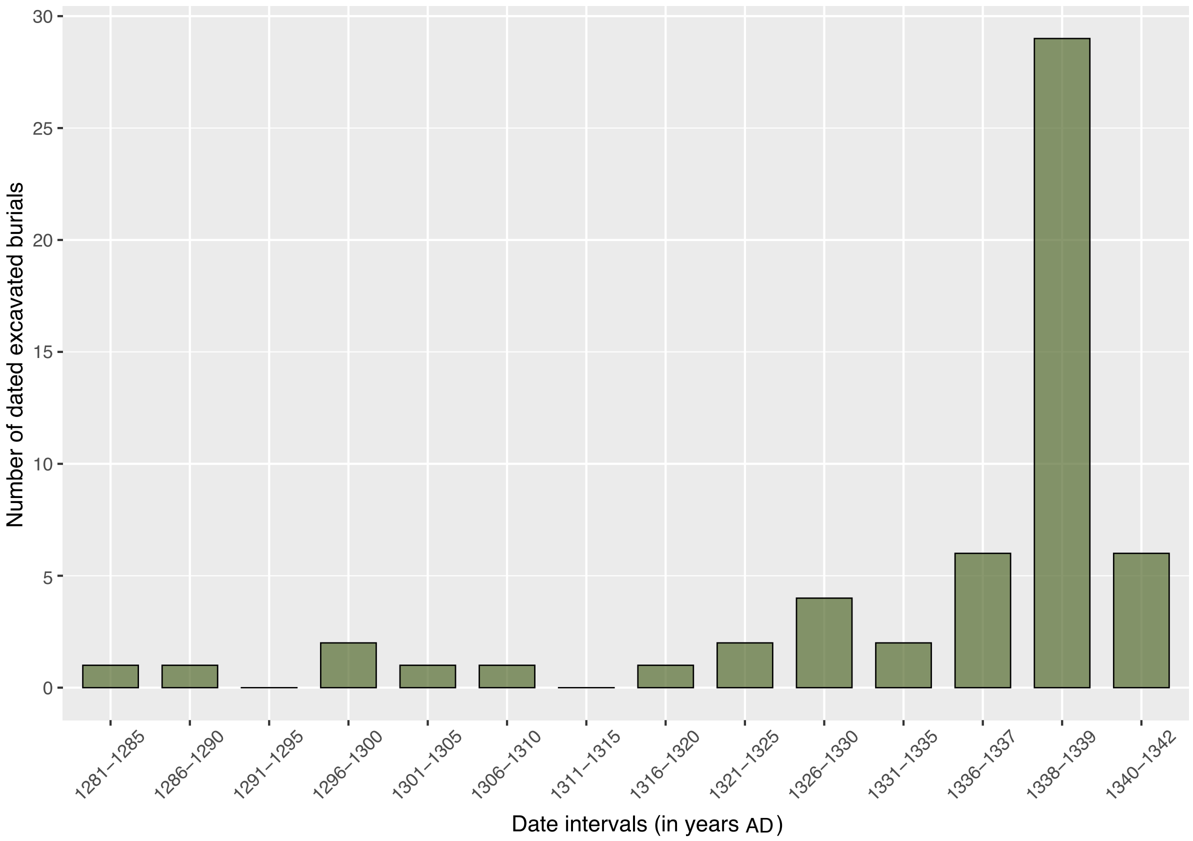

Supplementary Fig. 1 - Chronological distribution of dated Kara-Djigach burials, excavated in 1886 (dataset based on *AIIMKRAN, Fund 1, Inventory 1, File 40b, fols.2r-31r* and [18]).

Overall, the density of burials identified in Kara-Djigach was reported to be high. Specifically, the distance between burials was reported to be about 1 *sazhen'* (=2.13 m), while the distance between the burials and tombstones was between 1 and 1.5 *arshins* (71-107 cm) (*AIIMKRAN, Fund 1, Inventory 1, File 40b, fols.42r-45r*). The contents of each burial were recorded by N. Pantusov in his excavation diary, including skeletal heights, burial depths, coffin lengths and objects (*AIIMKRAN, Fund 1, Inventory 1, File 40b, fols.2r-31r*). Of the 90 skeletons excavated in the year 1886, ten can be identified as infants and 33 as sub-adults. As the original inventory does not include any specific identification of adult individuals, 47 adults were deduced here based on the indicated skeletal heights. Among the adults, on the basis of their associated tombstones and heights, 23 can be identified as males and 24 as females. Artefacts retrieved included pearls (Graves 9, 15, 26, 31, 35, 36, 61), corals (Grave 12), *Marginella* shells (Grave 18), precious metals and golden brocade cloths (Grave 43c), stone beads (Graves 8, 12, 17-18, 36, 38, 76), silk textiles (Grave 77) as well as a saddle and a bow (43c). Out of the total 83 burials unearthed in the course of the 1886 excavations, 56 can be precisely dated on the basis their associated tombstones. Of those, 29 were dated to the plague years, AD 1338-1339 (Supplementary Fig. 1). The highest concentration of graves associated with the plague years is found in rows 3-5, to the south and south-east of 'Chapel 1' (see Fig. 1 in the main text).

During the 1886 excavations, about 160 stones (mostly in Syriac, but some in Turkic) were collected and transferred to Verny (Almaty)[18] (*AIIMKRAN, Fund 1, Inventory 1, File 40b,*

fols.43v, 78r). Of these, 116 were sent to the *IAC* in late 1887. The *IAC*, in turn, donated the tombstones to the *Asiatic Museum of the Russian Academy of Sciences* (*AMRAS*) in St. Petersburg (*AIIMKRAN, Fund 1, Inventory 1, File 40b, fols. 80r, 81r, 82r, 84r*). In 1938, the stones were transferred from the *Museum of the History of Religion and Atheism* (the *AMRAS*'s successor since 1932) to the *Hermitage (Hermitage SA)*[76]. A further 11 stones were sent to the *Moscow Imperial Archaeological Society*, and the rest seem to have been retained in Verny (Almaty)[73].

Finally, in the course of the 1892 excavations (September-October 1892), ~350 additional tombstones (mostly in Syriac, but some in Turkic) were collected from the Kara-Djigach cemetery. That season did not involve any burial excavations[77]. In contrast with the tombstones collected in 1885 and 1886, the vast majority seem to have remained in Verny (Almaty), though with some exceptions. In June 1896, twenty of the excavated tombstones were sent to the Ministry of Public Education of the Third French Republic, which, in turn, deposited thirteen of these at the Guimet Museum in Paris[78,79]. A portion of those were thereafter moved to the Lyon branch of Guimet Museum. Currently, the whereabouts of only six tombstones deposited in France can be established, all in Lyon[71]. Moreover, in 1896, two tombstones were handed over to two central Eurasian explorers from Finland, Baron Carl Munck and Magister Otto Donner, who, in turn, brought them to Helsinki, where they are currently deposited at the Museum of Cultures[71,80]. Overall, the locations of only 24 stones from the 1892 expedition can be established at present.

Tombstone dataset from Kara-Djigach

The complete tombstone dataset from Kara-Djigach analysed for this study was compiled from sources listed as ref.[18,19,64,71,73,76,79-88]. This dataset includes c. 600 tombstones that were retrieved during the fieldwork seasons of 1885 (35 tombstones), 1886 (~160 tombstones) and 1892 (~350 tombstones), as detailed in the previous section, and also includes ~50 tombstones that were found and published ever since. Of those, a total 456 tombstones can be precisely dated on the basis of their associated inscriptions (Supplementary Table 1). Inscriptions on tombstones are dated on the basis of the Seleucid calendar, which East Syriac communities were using. The Seleucid year began from 1st October and ran until 30th September. The Seleucid era began on the 1st of October 312 BC, with Seleucus I Nicator's (one of Alexander the Great's generals) re-conquest of Babylon. The majority of tombstones are also dated according to the twelve-year animal-cycle, used by local Turkic peoples. A distribution of the annual numbers of tombstones, dated between AD 1248 and 1345, can be found on Fig. 1c of the main text and in Supplementary Table 1. Examples of available pictures of tombstones from Kara-Djigach associated with the individuals investigated in this study can be found in Extended Data Fig. 1.

Of 456 tombstones retrieved from Kara-Djigach, 114 are dated to the plague years of 1338-9 (Supplementary Table 1); ten of which contain plague inscriptions[37]. From those, photographs from two could be accessed and are shown in Extended Data Fig. 1d, e. These were captured by Abram S. Leibin, a professional photographer based in Verny (Almaty), hired by Nikolai

N. Pantusov (the supervisor of the Kara-Djigach excavations) for the duration of August 1886 excavation. In May 1887, the photographs were sent (alongside with 19 additional tombstone photographs) to the *Imperial Archaeological Commission*, which, in turn, shared them with D. Chwolson. The original Syriac texts of the inscriptions are printed in[18]. The current whereabouts of the two original tombstones are unknown.

Transliterations of the two pestilence-stating inscriptions are as follows:

Grave 30 (see Extended Data Fig. 1e):

'It was in the Year 1650 [=AD 1338-9], the Year of the Hare, in Turkic Taviškan. And this is the tomb of Qutluq. He died of plague [=*mawtānā*], with his wife, Mengü Qalqa'

Second stone, burial likely never excavated (lower left picture, Extended Data Fig. 1d):

'In the Year 1649 [=AD 1337-8], and it was the Year of the Tiger, in Turkic Bars. This is the tomb of the believer Sanmaq. [He] died of pestilence [=*mawtānā*]'

Kara-Djigach numismatic evidence

A further light on Kara-Djigach is shed by numismatic evidence, deriving from two Chaghadaid (Mongol)-period coin hoards, unearthed in the cemetery's vicinity, at later points[89,90]. The first assemblage, published in 2002, included 129 *dinars* and 10 *dirhams* (139 coins in total), while the second one, published in 2016, contains 20 *dirhams* and six *dinars* (26 coins in total). Of the total 165 coins, the Bukhara mint yielded 55 coins, that of Termez (the Uzbekistan-Afghanistan border) 47, the Samarqand mint 31, the Otrar mint (south Kazakhstan) 16. The Badakhshan mint (north-eastern Afghanistan) yielded three coins, those of Shash (Tashkent) and Almaliq yielded two each, while those of Taraz (on the Kazakhstan-Kyrgyzstan border) and Soltaniyeh (the capital of the Ilkhanid state, in north Iran) one each. The Badakhshan mint (north-eastern Afghanistan) yielded three coins, those of Shash (Tashkent) and Almaliq two each, while those of Taraz (on the Kazakhstan-Kyrgyzstan border) and Soltaniyeh (the capital of the Ilkhanid state, in north Iran) one each. The fact that some of these coins come from distant mints, some of which are situated thousands of kilometres away, suggests that long-distance trade was an established practice among the Kara-Djigach residents. Specifically, the present evidence suggests that this community had trade connections with regions as far away as Balkh, Badakhshan and northern Iran, situated up to 3,000 km south-west of Kara-Djigach. The chronological distribution of the two hoards is shown in Supplementary Fig. 2. As the figure shows, the latest identified coin dates to AD 1340-1341 (741 AH according to the Islamic Hijri calendar), which might indicate a possible abandonment of the settlement after that time, also reflected in the annual chronology of tombstones (see Fig. 1c of the main text).

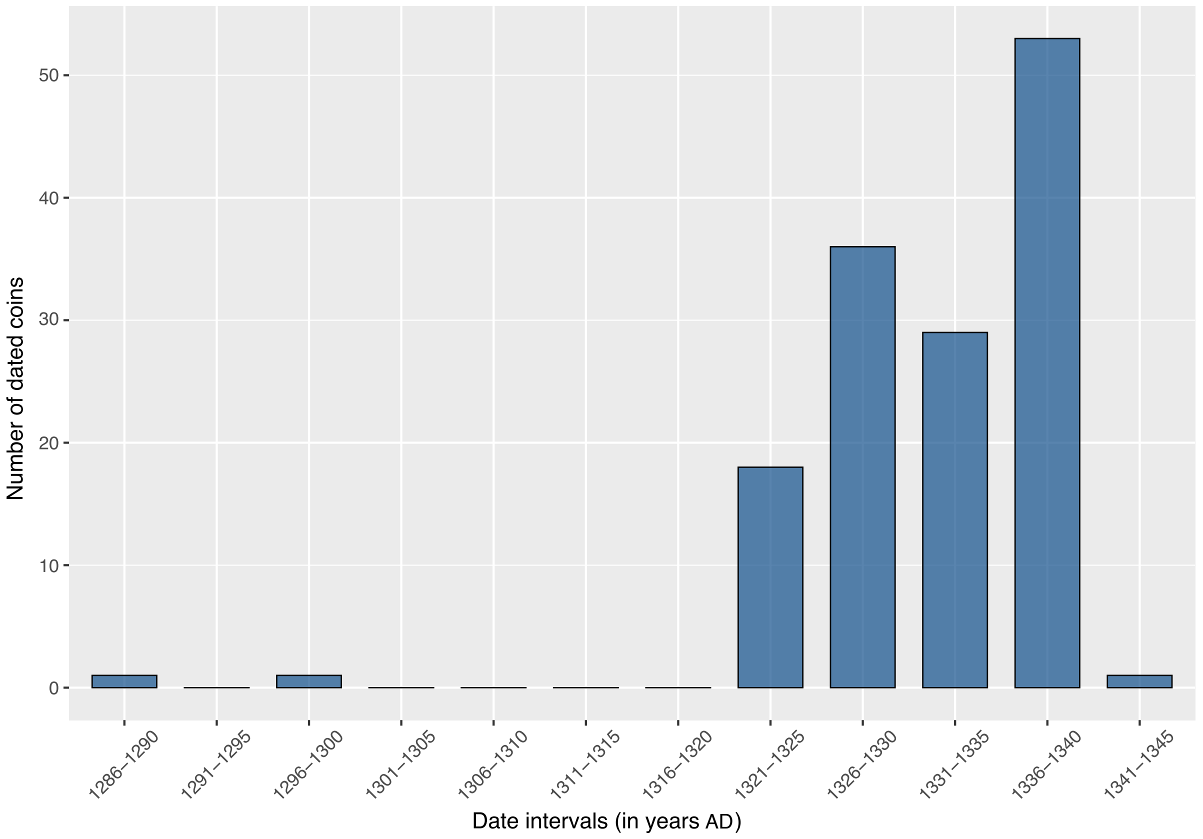

Supplementary Fig. 2 – Distribution of Kara-Djigach Chaghadaid-era coin hoards between AD 1286 and 1345 (as described in ref.[89,90])

Information on individuals from Kara-Djigach analysed in this study

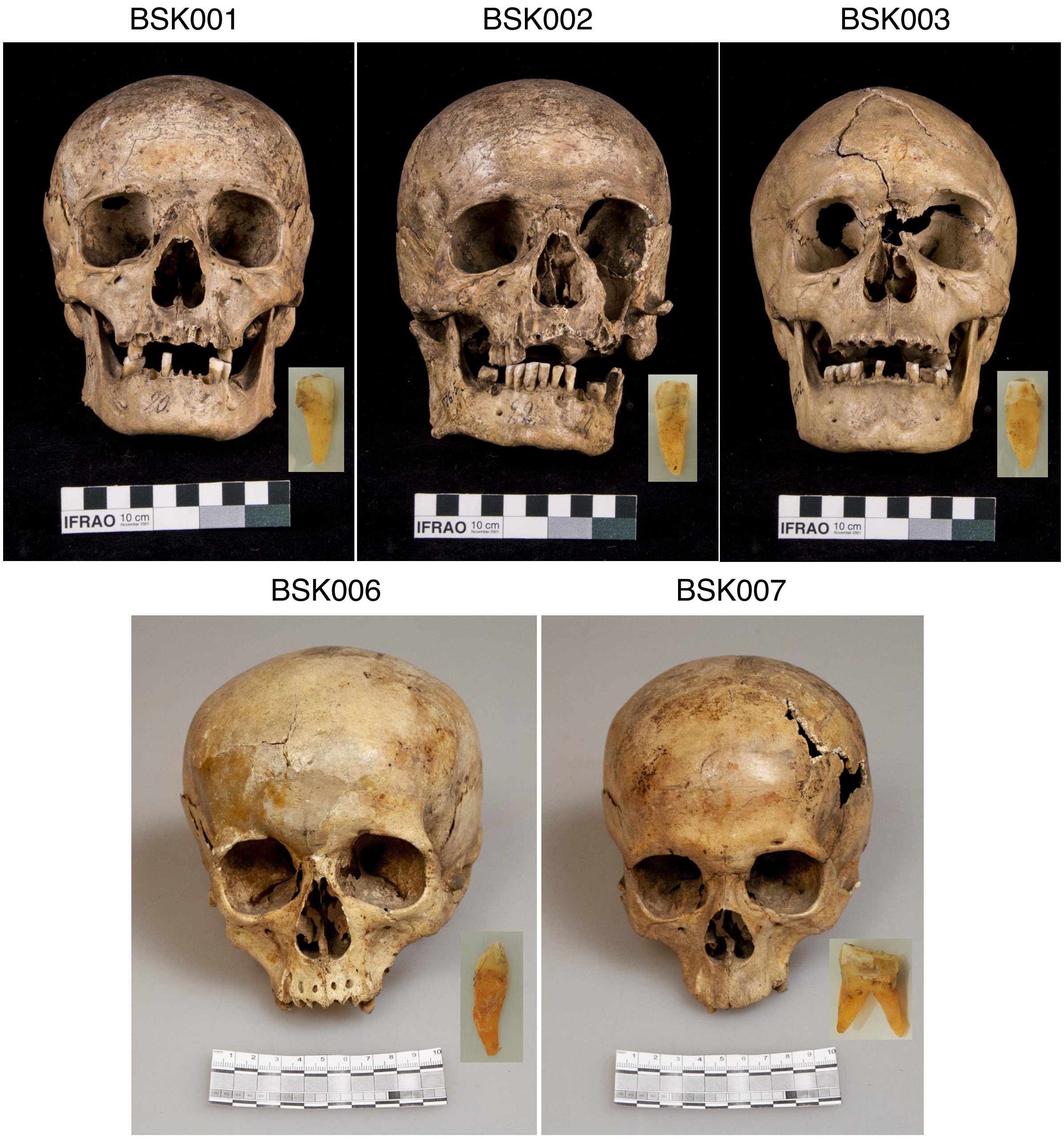

Supplementary Fig. 3 – Pictures of skulls and their associated tooth specimens from individuals buried in Kara-Djigach that were analysed in this study. From the collection of the Peter the Great Museum of Anthropology and Ethnography (*Kunstkamera*), Russian Academy of Sciences ©MAE RAS 2021 (MAE No. 176-4, 176-5, 176-7, 5559-1, 5559-2).

BSK001

The analysed tooth specimen belonged to an individual buried in Grave 20, which together with the adjacent Grave 19, represent two conjoined burials, described by N. Pantusov as a 'family tomb' (*AIIMKRAN, Fund 1, Inventory 1, File 40b, fols.12r*). The skeleton height of both individuals was measured at 146.7cm. No artefact was identified within the burials. Both individuals were interred into a bricked pit, rather than individual coffins (full description in *AIIMKRAN, Fund 1, Inventory 1, File 40b, fols.12r-v*). Based on cranial suture closure, BSK001 was classified as *maturus* in age (40-50 years).

It should be noted that the inscription initially associated with Grave 20 states "In the Year 1650 [=AD 1338-9], [it was the year of the] Hare. This is the tomb of Yoḥannan, a faithful man". However, the human genetic analysis associated with specimen BSK001 revealed the individual to be of female genetic sex (see Supplementary Table 3), which raises uncertainty regarding the initial tombstone identification or documentation. Moreover, a 1948 study of 17 skulls specimens from Kara-Djigach by G. Debets, identified the individual from Grave 20 as female[74]. Most likely, this discrepancy derives from the fact that the above-mentioned inscription is to be associated with Grave 19 and not 20. Conversely, the correct inscription associated with Grave 20 is that initially associated with Grave 19. Indeed, the tombstone inscription associated with Grave 19 states "In the Year 1650 [=AD 1338-9], [it was the year of the] Hare. This is the tomb of Bačaq, a faithful woman". Importantly, N. Pantusov described the skull from Grave 19 as 'decayed and disintegrated', which does not reflect the currently preserved state of the skull from Grave 20 (Supplementary Fig. 3), implying that the skulls, unlike headstones, were not mixed up. The fact that Graves 19-20 are a family burial may explain the erroneous inversion of the graves and their associated tombstones during initial *in situ* documentation during the August 1886 excavations. Alternatively, it is possible that the tombstones may have been inversely misplaced during the burial process at the time of the 1338-9 epidemic.

If this interpretation of the erroneous inversion of the graves and their associated tombstones is correct, then it appears that the analysed tooth specimen associated with the skull from Grave 20 belongs to a woman named Bačaq. The skull associated with this individual is currently held at the *Kunstkamera* and its accession reference number is 176/4 according to the hand-written catalogue of the museum's craniological collections (by Ye. Zhirov), file 176 (previous accession number 970 according to ref.[72]). The transition of the skull from the Kara-Djigach cemetery into the *IAC* in the year 1888, and its subsequent deposition at the *Kunstkamera* are documented in *AIIMKRAN, Fund 1, Inventory 1, File 40b, fols.*12r-v, 84v-85r, 119r. The original Syriac texts of the inscriptions of both burials within Grave 19-20 are printed in ref.[18]. The original stone associated with Bačaq's grave is at the *Hermitage* (*Hermitage SA no. 14432*). The current whereabouts of the stone associated with the male individual, Yoḥannan, are unknown.

BSK002

The analysed tooth specimen likely belonged to a young man, interred in Grave 22 (single burial). The man's name was Sargis, described as a 'student' (*eskolāyā* in Syriac) and he was interred into a bricked pit, with the skull positioned on an unfinished brick. The skeleton's height was measured at 153.4 cm. Based on cranial suture closure, BSK002 was classified as *maturus* (40-50 years) in age. No artefacts were described or identified within the grave. The skull associated with this individual is currently held at the *Kunstkamera* and its reference number is 176/5 according to the hand-written catalogue of the museum's craniological collections, file 176 (previous accession number 972 according to ref.[72]). The transition of the skull from the Kara-Djigach cemetery into the *IAC* occurred in the year 1888, and its subsequent deposition at the *Kunstkamera* is documented in *AIIMKRAN, Fund 1, Inventory 1, File 40b, fols.13r, 84v-85r, 119r*. In addition, the full description of the grave by N. Pantusov is in *AIIMKRAN, Fund 1, Inventory 1, File 40b, fol.13r*. A tombstone inscription associated with the grave states the following: "It was in the Year 1650 [=AD 1338-9], the year of the Hare, in Turkic Taviškan. This is the tomb of Sargis the student" (see ref. [18] for the original Syriac text of the inscription). The current location of the original stone is unknown.

It should be noted that the inscription above was initially associated with Grave 21, and not Grave 22[18]. In the cited edition, the inscription associated with Grave 22 refers to the burial of an adult woman called Qamta (most probably a mistake for Qyamta)[18]. However, the human genetic analysis associated with specimen BSK002 revealed the individual to be of male genetic sex (see Supplementary Table 4), which raises uncertainty regarding the initial tombstone identification or documentation. Moreover, a 1948 study of 17 skulls specimens from Kara-Djigach by G. Debets, identified the individual from Grave 22 as male and that from Grave 21 as a female[74]. Therefore, it is plausible that the information from Graves 21 and 22 was erroneously inversed during initial *in situ* documentation of the tombstones during the August 1886 excavations.

BSK003

The analysed tooth specimen belonged to an adult individual buried in Grave 28 (name illegible). Based on the genetic sex determination performed here, the individual was identified as a female (Supplementary Table 3). She was buried next to her relative (name illegible), likely her husband, buried in Grave 29. Based on cranial suture closure, BSK003 was classified as *maturus* (40-50 years) in age. The measured height of the skeleton within Grave 28 was 151.1cm. The female was not associated with any grave artefacts, whereas the individual of Grave 29 was buried with a golden ring, a ring with a precious stone and a bracelet. Both skeletons were described by N. Pantusov as 'eaten by worms' (indicated by fossilised larvae). The full description of the grave by N. Pantusov is in *AIIMKRAN, Fund 1, Inventory 1, File 40b, fols. 14r-v*. The skull associated with Grave 28 is currently held at the *Kunstkamera* and its accession reference number is 176/7 according to the hand-written catalogue of the museum's craniological collections, file 176. The transition of the skull from the Kara-Djigach cemetery to the *IAC* in the year 1888, and its subsequent deposition at the *Kunstkamera* are

documented in *AIIMKRAN, Fund 1, Inventory 1, File 40b, fols.12r-v, 84v-85r, 119r*. An only partially legible tombstone inscription associated with Graves 28 and 29 states: "In the Year 1650 [=AD 1338-9] … Hare, in Turkic Taviškan". This legible part of the original Syriac inscription is translated (into Russian) and printed in[73].

A direct radiocarbon date was generated for individual BSK003. Despite a wide temporal interval spanning the 13th and 14th centuries, the retrieved ^{14}C date falls within the described archaeological context (MAMS 52864; 708yr BP ±21; 95% probability calAD 1272-1378).

BSK006

The analysed tooth specimen was retrieved from Grave 6, where an adult woman named Bačaq was buried next to two young children and one infant. Based on cranial suture closure, BSK006 was classified as *senilis I* (over 60 years) in age. The adult woman was interred in the burial in woollen clothes and holding a metal ring on the right hand. The height the woman's skeleton was measured at 146.7 cm. The full description of the grave by N. Pantusov is in *AIIMKRAN Fund 1, Inventory 1, File 40b, fols. 6v-7r*. The skull associated with Grave 6 is currently held at the *Kunstkamera* and its reference number is 5559/1 according to the hand-written catalogue of the museum's craniological collections, file 5559 (previous accession number *LSU, FA*, no. 1196). The transition of the skull from the Kara-Djigach cemetery into the *IAC* in the year 1888 is documented in *AIIMKRAN, Fund 1, Inventory 1, File 40b, fols.6v-7r, 84v-85r, 119r*. The eventual transfer of the skull from the *LSU, FA* to *Kunstkamera* in the year 1937 is documented in the hand-written catalogue of the museum's craniological collections: file 5559. A tombstone inscription associated with Grave 6 states: "This is the grave of Bačaq, a faithful woman. Amen". The original Syriac text on the inscription is printed in[18]. The original stone is at the *Hermitage* (*Hermitage SA* no. 14428).

BSK007

The analysed tooth specimen belonged to a woman named Qutluq Tärim. She was buried in Grave 9 (single burial) alongside a bracelet on the left hand, a ring on the right hand and a golden earring with four pearls on the left ear. All such artefacts may indicate a higher socioeconomic status of this individual. Based on cranial suture closure, BSK007 was classified as *maturus-senilis* (50-60 years) in age. The height of the skeleton was estimated at 144.5 cm. The full description of the grave by N. Pantusov is in *AIIMKRAN, Fund 1, Inventory 1, File 40b, fols. 8r-v*. The skull associated with this individual is currently held at the *Kunstkamera* and its accession reference number is 5559/2 according to the hand-written catalogue of the museum's craniological collections: file 5559 (previous accession number *LSU, FA*, no. 1197). The transition of the skull from the Kara-Djigach cemetery into the *IAC* in the year 1888 is documented in *AIIMKRAN, Fund 1, Inventory 1, File 40b, fols. 8r-v, 84v-85r, 119r*. The eventual transfer of the skull from the *LSU, FA* to *Kunstkamera* in the year 1937 is documented in the hand-written catalogue of the museum's craniological collections: file 5559. Grave 9 is associated with a tombstone that includes the following inscription: "In the Year 1650 [=AD 1338-9], and it was the Year of the Hare, in Turkic Taviškan. And this is the

tomb of Qutluq Tärim, a faithful woman". The original Syriac text of the inscription is printed in[18]. The original stone is at the *Hermitage* (*Hermitage SA no. 14352*).

A direct radiocarbon date was produced for individual BSK007. Despite a wide temporal interval spanning the 13th and 14th centuries, the retrieved ^{14}C date falls within the described archaeological context (MAMS 52865; 701yr BP ±20; 95% probability calAD 1275-1379).

The Burana cemetery

Individuals analysed:

- BSK004 (188/1; Grave IV)
- BSK005 (188/2; Grave X)

Background information on the cemetery and 19th century excavations

The cemetery was located ~1.5 km to the south of the modern-day village of Burana, 10 km south of the town of Tokmok. During the Qarakhanid, Qara-Khitai and Mongol times, Burana was a major city, identified by some with the City of Balasaghun, the capital of Qara-Khitai Khanate. It is difficult to estimate the complete area occupied by the medieval Christian cemetery, but given the low number of excavated graves and tombstones, it was likely smaller than the Kara-Djigach cemetery. In the course of the 1885 and 1886 excavations, two areas were excavated: one ~1.6 km to the south of the Burana tower (approximate coordinates: N42°42'59.95" E75°14'34.88") and the second to the immediate north-west of the tower (approximate coordinates: N42°44'50.73" E75°14'57.26").

The cemetery was first discovered in October 1885 by a local resident and medicine practitioner, Dr. F. V. Poyarkov. In the course of the excavations conducted by Poyarkov, 16 graves containing 16 skeletons were excavated, of which seven human skulls were removed. In addition, 22 tombstones (most in Syriac, but some in Turkic) were retrieved. Apart from some photographic documents, the outcome of the 1885 excavation was not recorded. Instead, the findings were reported at the annual meeting of the *Historical-Philological Division of the Imperial Academy of Sciences* session in April 1886 by N. M. Yadrintsev, a St-Petersburg-based explorer and archaeologist[91]. In addition, tombstone photographs were made available to D. Chwolson of St. Petersburg University, who published five of the tombstones[64]. The current location of the 16 skeletons and 22 tombstones excavated by Poyarkov are unknown.

The second season of excavations at Burana, headed by A. Fetisov, took place in August 1886 (*AIIMKRAN, Fund 1, Inventory 1, File 40b, fols.102r-122v*). In the cemetery, the distance between graves was reported to be only about one metre. Overall, the Burana excavation yielded 33 graves containing 34 skeletons. Of 34 skeletons, 20 were identified as sub-adults. Overall, sub-adult burials were also reported to have more shallow depths (1.2-1.4 metres), compared to the average depth of 1.6-2 metres for adult burials. The contents of each grave were recorded by A. Fetisov, with artefacts retrieved including pearls (Grave XXI), corals (Grave XXX), stone beads (Graves XXV, XXX-XXXII), precious metals and golden brocade

cloths (Graves XIX and XXXI) (*AIIMKRAN, Fund 1, Inventory 1, File 40b, fols.102r-122v*). Of the recovered skeletal assemblage, six human skulls (Graves IV, X, XI, XII, XIV, XX) were sent to St. Petersburg in June 1888, to the charge of the *IAC*, which moved them shortly after that to the *Kunstkamera* (*AIIMKRAN, Fund 1, Inventory 1, File 40b, fols.74r-v*). At present, only four skulls from Burana remain at the *Kunstkamera* according to Ye. Zhirov's handwritten catalogue of the museum's craniological collections: file 188 and ref.[74]. Finally, as at Kara-Djigach, the 1892 excavations season (September-October 1892), did not involve any burial excavations.[77]

Tombstone dataset from Burana

In contrast with the Kara-Djigach cemetery, Burana yielded much fewer inscriptions. In addition to 22 tombstones discovered by Poyarkov in October 1885, only nine inscriptions (mostly in Syriac, but some in Turkic) were found in the course of the August 1886 excavations and a further five during the Autumn 1892[77] (*AIIMKRAN, Fund 1, Inventory 1, File 40b, fol. 149r*). No graves were dug in the course of the 1892 excavations. The Burana tombstones confirm that most burials relate to sub-adults, by specifying the Syriac age-associated terms *ṭalyā/ṭlītā* (boy/girl usually indicating the age of under twelve) and *ʿlaymā/ʿlaymtā* (young man/maiden, usually indicating the age range of twelve to 20)[18,19,64]. Of the total 36 tombstone inscriptions collected in the course of the 1885, 1886 and 1892 excavations, only 14 were published by D. Chwolson[18,19,64], and a further eleven were found and published even since[76,81,82]. Of those, only eleven can be precisely dated with four being from the years AD 1338-1339 (Supplementary Table 1)[19,76]. It should be noted that, in contrast to the Kara-Djigach cemetery, many tombstones from Burana feature only a cross, without inscriptions – a characteristic of sub-adult burials.

Information on the individuals from Burana analysed in this study

BSK004 BSK005

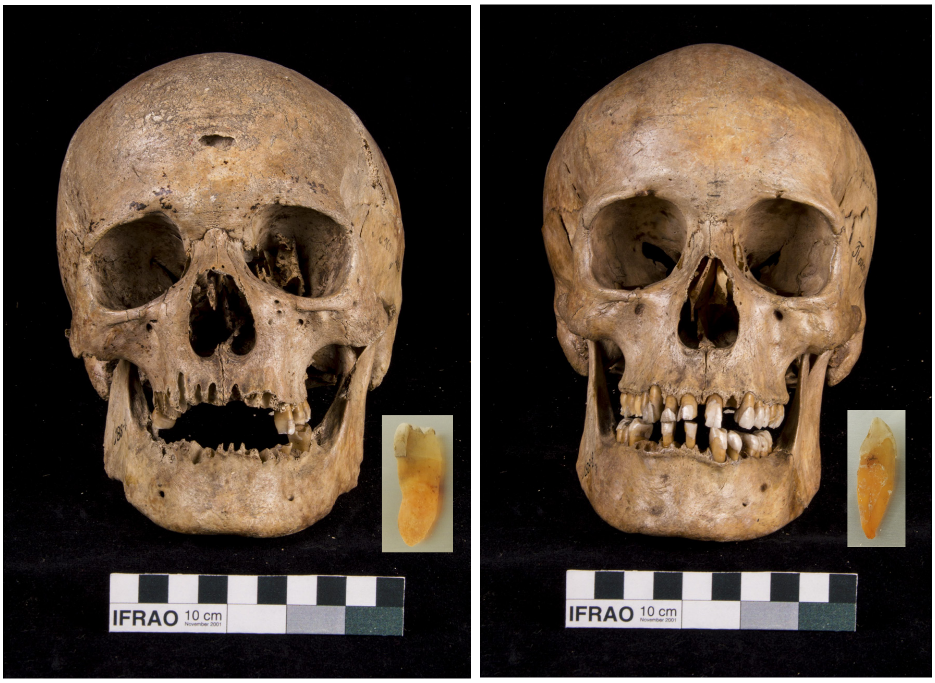

Supplementary Fig. 4 - Pictures of skulls and their associated tooth specimens from individuals buried in Burana that were analysed in this study. From the collection of the Peter the Great Museum of Anthropology and Ethnography (*Kunstkamera*), Russian Academy of Sciences ©MAE RAS 2021 (MAE No. 188-1, 188-2).

<u>BSK004</u>

The analysed tooth specimen belonged to an adult individual. Based on the genetic analysis performed in this study, the individual is identified as a male. Based on cranial suture closure, BSK004 was classified as *maturus* (40-50 years) in age. The skull is currently held at the *Kunstkamera* and its accession reference number is 188/1 according the hand-written catalogue of the museum's craniological collections: file 188 (previous accession number 1012 according to ref.[72]). The individual, buried in Grave IV (single burial) was interred into a bricked pit, with no coffin, and the length of the burial was measured at 195.6cm. No tombstone was found in association with this burial. The full description of the grave by A. Fetisov can be found in *AIIMKRAN, Fund 1, Inventory 1, File 40b, fols.*105v-106r. The transition of the skull from the Burana cemetery into the *IAC* in 1888, and its subsequent deposition at the *Kunstkamera* is documented in *AIIMKRAN, Fund 1, Inventory 1, File 40b, fols.*13r, 84v-85r, 119r.

<u>BSK005</u>

The analysed tooth specimen was unearthed from Grave X containing the remains of an individual, potentially a woman, interred into the burial without a coffin. Based on cranial suture closure, BSK005 was classified as *adultus-maturus* (30-40 years) in age. The height of

the associated skeleton was measured at 151.1 cm. A later assessment of the individual's skull morphology led to a female sex assignment[74]. The skull is currently held at the *Kunstkamera* and its accession reference number is 188/2 according to the hand-written catalogue of the museum's craniological collections: file 188 (previous accession number 1013 according to ref.[72]). No artefacts associated with this burial were described or taken. In addition, a child's skeleton, was identified over Grave X, designated as Grave IX, possibly indicating a familial relationship between the two individuals. No tombstone was found in association with either of the burials. The full description of the burials by A. Fetisov can be found in *AIIMKRAN, Fund 1, Inventory 1, File 40b, fols.* 107r-108r.

Toponymic indications of mobility in Kara-Djigach and Burana

The profile of the Kara-Djigach Christian community can partly be gleaned from both inscriptions and associated archaeological finds. Across the entire chronology of excavated tombstones (1248-1345) (Supplementary Table 1), Turkic personal names accounted for almost two-thirds of all names, with the remaining consisting of Biblical Hebrew and Greek names, in their Syriac forms[18,19,92]. As William de Rubruck noted while travelling to the Qa'an's court in Karakorum during AD 1253-1255, local Christians prayed in Syriac without knowing this language[65]. These facts suggests that Kara-Djigach, and other Semirech'ye Christian communities, likely consisted of native Turkic speakers.

Toponymic evidence identified among the tombstones of Kara-Djigach and Burana contributes important information on the population diversity of the Chüy Valley region. Although the epitaphs do not mention the ethno-linguistic background of most interred individuals, they mention at least one individual designated as a 'Mongol', one as 'Uyghur', one as 'Armenian' and two as 'Chinese' (see inscription translations below). In addition, there are eight inscriptions with toponymic surnames, with seven individuals designated as 'of Almaliq' (*Almāligayā*/*Almāligaytā*), and one individual bearing the surname *Kaškarayā* ('of Kashgar'). Both cities were among some of the largest and most important centres along long-distance trans-Asian trade routes, and homes to different ethnic, linguistic, and religious groups, including East Syriac Christian communities, during the 13th and 14th centuries[61,86]. Taken together, it appears likely that the East Syriac communities of Kara-Djigach and Burana had a diverse profile where mobility from other parts of Semirech'ye and further-away regions of central Eurasia played an important role in their makeup. Although immigration played a particularly important role in the earlier days of the East Syriac community of Kara-Djigach (and presumably, Burana, too) between c.1250 and 1300, later stones (nos. 9-11 below) indicate that this migration continued as the time passed. Below are all the known/surviving epitaphs associated with toponymic attributes:

1. Provenance: Kara-Djigach; text: "It was in the Year 1597 [=AD 1285-6] (the Year of the) Dog. This is the tomb of the faithful woman Tärim the Chinese (Tārīm Ṣīnītā)" Source: Chwolson, D. *Syrisch-nestorianische Grabinschriften aus Semirjetschie, neue Folge*. (Commissionnaires de l'Académie impériale des sciences, 1897), no. 24[19]

2. <u>Provenance</u>: Kara-Djigach; <u>text</u>: "It was in the Year 1598 [=AD 1286-7], the Year of the Pig. And this is the tomb of the Priest George of Almaliq (Gyorgīs qašā Almaligāyā)"
   Source: Chwolson, D. Syrisch-nestorianische Grabinschriften aus Semirjetschie. *Mémoires de l'Académie Impériale des Sciences de St.-Pétersbourg. VII ser.* XXXVII:8 (1890), no. 98 (52)[18]

3. <u>Provenance</u>: Kara-Djigach; <u>text</u>: "It was in the Year 1599 [=AD 1287-8], the Year of the Rat. And this is the tomb of Tabita of Almaliq, faithful woman (Ṭabītā mhaymantā Almaligāytā)"
   Source: Chwolson, D. Syrisch-nestorianische Grabinschriften aus Semirjetschie. *Mémoires de l'Académie Impériale des Sciences de St.-Pétersbourg. VII ser.* XXXVII:8 (1890), no. 99 (116)[18]

4. <u>Provenance</u>: Kara-Djigach; <u>text</u>: "It was in the Year 1603 [=AD 1291-2], the Year of the Dragon. And this is the tomb of the Sargis of Almaliq (Sargīs qašīšā Almaligāyā)"
   Source: Chwolson, D. Syrisch-nestorianische Grabinschriften aus Semirjetschie. *Mémoires de l'Académie Impériale des Sciences de St.-Pétersbourg. VII ser.* XXXVII:8 (1890), no. 3,3 (196)[18]

5. <u>Provenance</u>: Kara-Djigach; <u>text</u>: "It was in the Year 1603 [=AD 1291-2], the Year of the Dragon. And this is the tomb of Yohannan (John) the chorepiscopus of Almaliq (Yoḥannān korappesqōpā Ṣyāmarṭ Almāligayā)"
   Source: Chwolson, D. Syrisch-nestorianische Grabinschriften aus Semirjetschie. *Mémoires de l'Académie Impériale des Sciences de St.-Pétersbourg. VII ser.* XXXVII:8 (1890), no. 3,5 (196,a)[18]

6. <u>Provenance</u>: Kara-Djigach; <u>text</u>: "It was in the Year 1603 [=AD 1291-2], (the year of the) Dragon. And this is the tomb of the Priest Yohannan (John) the Tapqač (Chinese) (Yoḥannān qašā Tābgāṣ)"
   Source: Chwolson, D. Syrisch-nestorianische Grabinschriften aus Semirjetschie. *Mémoires de l'Académie Impériale des Sciences de St.-Pétersbourg. VII ser.* XXXVII:8 (1890), no. 3,1 (121)[18]

7. <u>Provenance</u>: Kara-Djigach; <u>text</u>: "It was in the Year 1611 [=AD 1299-1300], the Year of the Rat. And this is the tomb of the Priest Moses of Almaliq (Mūšē qašīšā Almaligāyā), who was eagerly dedicated to the work of Church"
   Source: Chwolson, D. Syrisch-nestorianische Grabinschriften aus Semirjetschie. *Mémoires de l'Académie Impériale des Sciences de St.-Pétersbourg. VII ser.* XXXVII:8 (1890), no. 11,2 (144)[18]

8. <u>Provenance</u>: Kara-Djigach; <u>text</u>: “It was in the Year 1611 [=AD 1299-1300], the Year of the Rat. And this is the tomb of the Priest George of Almaliq (Gyorgīs qašā Almaligāyā)”
   Source: Chwolson, D. Syrisch-nestorianische Grabinschriften aus Semirjetschie. *Mémoires de l'Académie Impériale des Sciences de St.-Pétersbourg. VII ser.* XXXVII:8 (1890), no. 11,1 (59)[18]

9. <u>Provenance</u>: Kara-Djigach; <u>text</u>: [In Armenian]: “Christ Jesus, Lord God. Sir Yovan (John), Armenian Bishop. This commemoration has been written in the Year 772 [=AD 1322-3] of the Armenian Era” [In Syriac]: “This is the grave of Yohannan (John), Armenian Bishop (Yoḥannān appesqopā armnayā)”
   Source: Chwolson, D. *Syrisch-nestorianische Grabinschriften aus Semirjetschie, neue Folge*. (Commissionnaires de l'Académie impériale des sciences, 1897), no. 100[19]; Marr, N. Nadgrobnyi kamen' iz Semirechiya s Armyanskoi-Siriiskoi Nadpis'yu 1323 g.. *Zapiski Vostochnago Otdeleniya Imperatorskago Russkago Arkheologicheskago Obshchestva* 8, pp. 344-349 (1894)[92]

10. <u>Provenance</u>: presumably, Kara-Djigach; <u>text</u>: “It was in the Year 1635 [=AD 1323-4 CE], the Year of the Rat. And this is the tomb of the Priest Banos the Uyghur (Banos qašā Uigur)”
    Source: Chwolson, D. *Syrisch-nestorianische Grabinschriften aus Semirjetschie, neue Folge*. (Commissionnaires de l'Académie impériale des sciences, 1897), no. 97[19]

11. <u>Provenance</u>: presumably, Kara-Djigach; <u>text</u>: “It was in the Year 1652 [=AD 1340-1 CE], the Year of the Snake, in Turkic‘Ilan’. And this is the tomb of Qyamta of Kashgar, faithful woman (Qyamtā mhaymantā Kaškaray[t]ā)”
    Source: Chwolson, D. *Syrisch-nestorianische Grabinschriften aus Semirjetschie, neue Folge*. (Commissionnaires de l'Académie impériale des sciences, 1897), no. 211[19]

12. <u>Provenance</u>: presumably, Kara-Djigach; <u>text</u>: (undated) “This is the grave of Qyamta of Almaliq, faithful woman (Qyamtā mhaymantā Almāligaytā)”
    Source: Chwolson, D. *Syrisch-nestorianische Grabinschriften aus Semirjetschie, neue Folge*. (Commissionnaires de l'Académie impériale des sciences, 1897), no. 261[19]

13. <u>Provenance</u>: presumably, Kara-Djigach; <u>text</u>: (undated) “This is the grave of Tata the Mongol, faithful man (Ţāṭā mhaymnā mogolayā)”
    Source: Chwolson, D. *Syrisch-nestorianische Grabinschriften aus Semirjetschie, neue Folge*. (Commissionnaires de l'Académie impériale des sciences, 1897), no. 268[19]

Supplementary Information 3

Human aDNA analysis

Methods

Human aDNA data processing

After 1240K capture enrichment, raw sequenced reads were processed through the EAGER 1.92.58 workflow[93]. AdapterRemoval v2.2.0 was used to clip the adaptors and discard reads shorter than 30 bp[94]. Genome mapping to Human Reference Genome Hs37d5 was done with BWA v0.7.12 aln/samse[95] setting the edit distance parameter (-n) to 0.01 and the seed length (-l) to 32. The program DeDup v0.12.2 was used to mark and remove PCR duplicates[93]. Finally, only reads with phred mapping quality ≥ 30 were retained using SAMtools v1.3[96], and considered for genotype calling.

Genetic sex was determined by calculating the ratio between the coverage on the X and Y chromosomes over the one on the autosomes[97]. Among the analysed individuals, three were assigned as female (BSK001, BSK003 and BSK005) and two as male (BSK002 and BSK004), whereas BSK006 and BSK007 were too low in coverage to assign genetic sex.

The program mapDamage v2.0[98] was used to estimate the levels of deamination at the ends of the fragments, that results in high proportions of C-to-T mismatches to the reference genome at the 5' end and the complementary G to A at the 3' end. All analysed specimens showed deamination levels between 5% and 20% at the first and last base, falling within the expected range for partially UDG-treated double-stranded libraries. Nuclear-based contamination with exogenous human DNA was estimated for male individuals based on the levels X-chromosome heterozygosity using ANGSD v0.910[99], whereas mitochondrial DNA (mtDNA) contamination was estimated for both male and female individuals with Schmutzi[100]. All analysed specimens showed negligible signs of contamination (highest estimate < 2%), however, contamination estimates for BSK006 and BSK007 could not be retrieved due to low coverage. Schmutzi was also used to obtain mtDNA consensus sequences with a mapping quality cut-off of 10, 20 and 30. HaploGrep2[101] was then used to assign haplogroups.

Genotyping and PCA

Pseudo-haploid genotype calls were done with pileupCaller using the --randomHaploid parameter to perform a random calling of only one high quality base within the 1240K panel (https://github.com/stschiff/sequenceTools). Transitions were called on reads after masking the first 2 bp at each end with trimBam module of bamUtil v.1.0.13[102]. The full-length reads were instead used to call transversions.

The newly produced genotype data were merged with a reference panel composed of 2280 modern individuals genotyped with the Human Origins array[103-105]. The estimated overlap between the newly analysed specimens and the Human Origins SNP panel ranged between

1,371 and 372,828 SNPs. Individuals BSK001, BSK002, BSK003 and BSK005 exhibited coverages that were confidently over the empirical thresholds usually considered (10,000 - 30,000 SNPs), while BSK004, BSK006 and BSK007 presented too few SNPs covered and were therefore excluded from the subsequent analyses.

This dataset consisting of 586,594 overall SNPs, was used for running principal component analysis with the function smartpca v16000 in EIGENSOFT v6.0.1 package[106]. The lsqproject (i.e. least square projection) option was used to project the data of the ancient individuals on top of the PCA calculated on the set of modern individuals. For this analysis we chose a panel of 150 present-day Eurasian populations, most of them representing small or isolated communities and/or individuals belonging to ethnic groups[103-105], in order to locate the genetic ancestry of ancient samples within the broad genomic landscape of Eurasia.

Genetic relatedness

We estimated genetic relatedness between the individuals calculating a commonly used statistics called pairwise mismatch rate (pMMR) which is the rate of mismatching alleles between each pair of individuals[107]. This statistic has resolution to identify genetic relatedness down to second degree relatives[107]. We also calculated a similar statistic implemented in READ[108] that estimates pMMR in windows of 100kb along the genome which allows to calculate standard errors for the estimated degrees of relatedness (identical, first and second degree). None of the individuals analyzed resulted to be close genetic relatives (Supplementary Table 3).

Ancestry modelling

We merged our data with a reference dataset downloaded from the version v50.0 of the Allen Ancient DNA resource (https://reich.hms.harvard.edu/allen-ancient-dna-resource-aadr-downloadable-genotypes-present-day-and-ancient-dna-data) consisting of previously published ancient and modern genome-wide data including the SNPs overlapping between the 1240K panel and the HumanOrigins micro array (597,573 SNPS). We then applied for all BSK individuals the ancestry deconvolution framework of *qpWave/qpAdm* (v1520) based on fitting multiple *f4*-statistic to test for feasible sources of ancestry in a target population or individual[109,110]. The method estimates standard errors (SE) of the ancestry proportions using a block jackknife approach with 5 centiMorgan (cM) blocks. We used the "allsnps: NO" parameter that makes use of the SNP overlap between all groups for each f4-test (the same set of SNPs is used for all the tests on the same target). We selected a set of outgroups that includes representatives of the main west, south and east Eurasian ancient lineages described so far and relevant representative non-Eurasian ancestries and a set of geographically relevant and temporal preceding groups as sources of admixture (Supplementary Tables 4, 5). In choosing the outgroups we used direct ancient sources when available preferring groups or individuals with higher coverage otherwise we selected present-day proxies of specific distal lineages: Mbuti, Jordan_PPNB, Turkey_N, Iran_GanjDareh_N, IronGates_Mesolithic, Russia_HG_Karelia, Onge, Mixe, DevilsCave_N.SG, Russia_Bolshoy, Russia_Kolyma_M.SG, China_YR_LN

Results

Human genomic results from Kara-Djigach and Burana

Human DNA enrichment yielded allele calls in >30,000 SNPs for individuals BSK001, BSK002, BSK003 and BSK005 individuals, while BSK004, BSK006 and BSK007 produced insufficient data for inclusion in further nuclear DNA analyses (Supplementary Table 3). Genetic-sex determination assigned both BSK001, BSK003 and BSK005 as female, whereas individuals BSK002 and BSK004 as male (Supplementary Table 3). Mitochondrial and Y-chromosome DNA analysis revealed haplogroups, which are common among historical and modern-day central Eurasian populations for individuals BSK001, BSK002, BSK003 and BSK004 (Supplementary Table 3)[111-114]. In addition, a genome-wide principal component analysis (PCA) computed with present-day Eurasian individuals, placed individuals BSK001, BSK002, BSK003 and BSK005 within the diversity of central Eurasian populations (Supplementary Fig. 5), though not directly overlapping with present-day ethnic groups from Kyrgyzstan. We performed individual based modeling of BSK001 (85,996 overlapping SNPs), BSK002 (293,486 overlapping SNPs), BSK003 (94,220 overlapping SNPs) and BSK005 (100,702 overlapping SNPs) and we also grouped together BSK001, BSK003 and BSK005 (Bishkek) given their proximity in PCA space to gain resolution given the higher SNP overlap (302,211 SNPs). Overall, the results of these models show that all individuals can be best modeled as a mixture of temporally preceding central Eurasian populations with a minor (~10%) genetic influx from eastern Eurasia (Supplementary Tables 4, 5). The major source best matches either 6th-century Alanic period groups (Alan) from the Pontic-Caspian steppe or 3rd-century southern Kazakhstan groups related to the Kangju culture (Konyr_Tobe_300CE or Kazakhstan_Kangju.SG)[113-116]. The minor source best matches older Iron Age Saka/Scythian groups in the case of BSK002 or eastern steppe 1st - 2nd century AD Xiongnu or Xianbei period groups[113-116] for BSK001, BSK003 and BSK005 (Supplementary Tables 4, 5).

The very limited ancient DNA data available from the 1st millennium AD onwards across central Eurasia makes the testing of finer scale hypothesis unfeasible. Nevertheless, these results are in agreement with the depicted PCA clustering, which shows that a broadly defined early medieval central Eurasian genetic source is to be preferred over other sources that instead provide only largely unfeasible models (Pvalue << 0.05; Supplementary Tables 4, 5).

Connections between the Kara-Djigach community and several regions across Eurasia are demonstrated through rich numismatic and material evidence, as well as through toponymic indications identified at the site (Supplementary Information 2, Supplementary Fig. 2). Nonetheless, previous studies have shown high levels of genetic heterogeneity among the peoples of central Eurasia during historical periods[113,114], and have suggested that the observed homogeneity of present-day groups formed within the last 500 years. As such, future investigations in the region will reveal whether the genetic profiles identified here were part of the early-14th century local gene-pool.

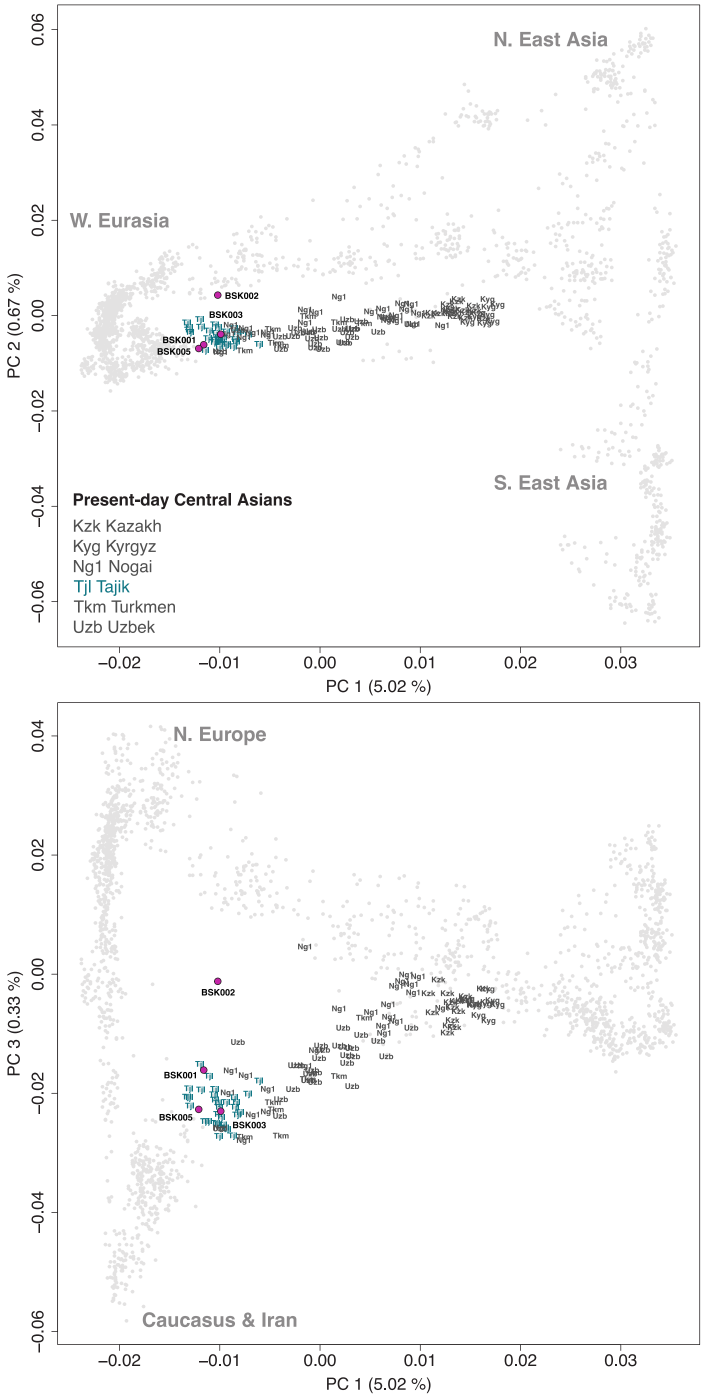

Supplementary Fig. 5 – Comparative human genome-wide SNP profiles of individuals BSK001, BSK002, BSK003 and BSK005. Principal component analysis (PCA) calculated on the genome-wide SNP data of present-day Eurasian populations on which ancient individuals are projected. The upper panel shows PC1 vs PC2 and the bottom panel shows PC1 vs PC3. Present day individuals are visualized with small grey circles. Dark grey labels indicate the approximate position of populations from the specified macro geographic regions. Present-day central Eurasian populations are instead visualized with a three-letter code detailed in the bottom left corner of the first plot. Colours refer to their language family: Turkic in grey and Indo-European (only Tajiks) in light blue.

Supplementary Information 4

Additional supplementary figures

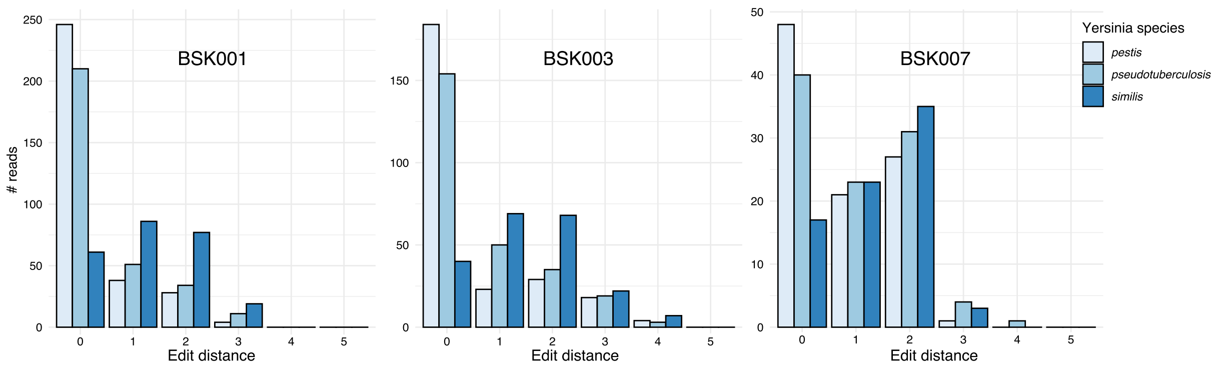

Supplementary Fig. 6 – Edit distance of shotgun sequenced reads mapping against members of the *Y. pseudotuberculosis* complex, *Y. pestis*, *Y. pseudotuberculosis* and *Y. similis*, in three individuals showing metagenomic signatures of ancient *Y. pestis* DNA. Reads mapping against *Y. pestis* and *Y. pseudotuberculosis* are showing overall declining edit distances. The majority of reads of lowest edit distance are found to be mapping against *Y. pestis* and, therefore, individuals BSK001, BSK003 and BSK007 were considered as putatively-positive and were subjected to whole-genome enrichment. All panels were created using the ggplot2[117] package on R version 3.6.1[118].

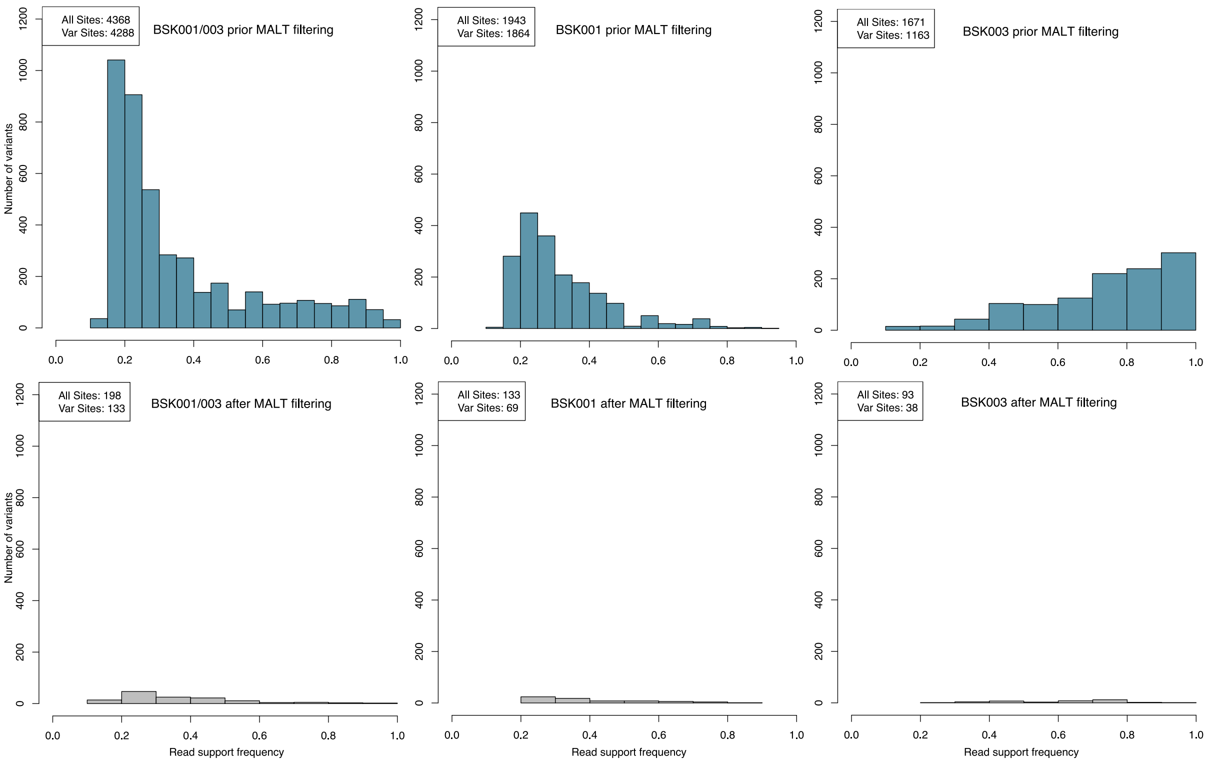

Supplementary Fig. 7 – Histograms of *Y. pestis* SNP allele frequencies in BSK001 and BSK003. The displayed histograms show the distribution of frequencies of alternative (non-reference) alleles across the AD 1338-1339 genomes BSK001 and BSK003 from Kara-Djigach. The histograms shown in blue are of all identified variants among reads mapping against the *Y. pestis* CO92 reference genome (NC_003143.1). The histograms shown in grey are the product of metagenomic taxonomy-based filtering, where reads assigned to the *Yersinia* node and summarised under the *Y. pseudotuberculosis*-complex node were exclusive selected.

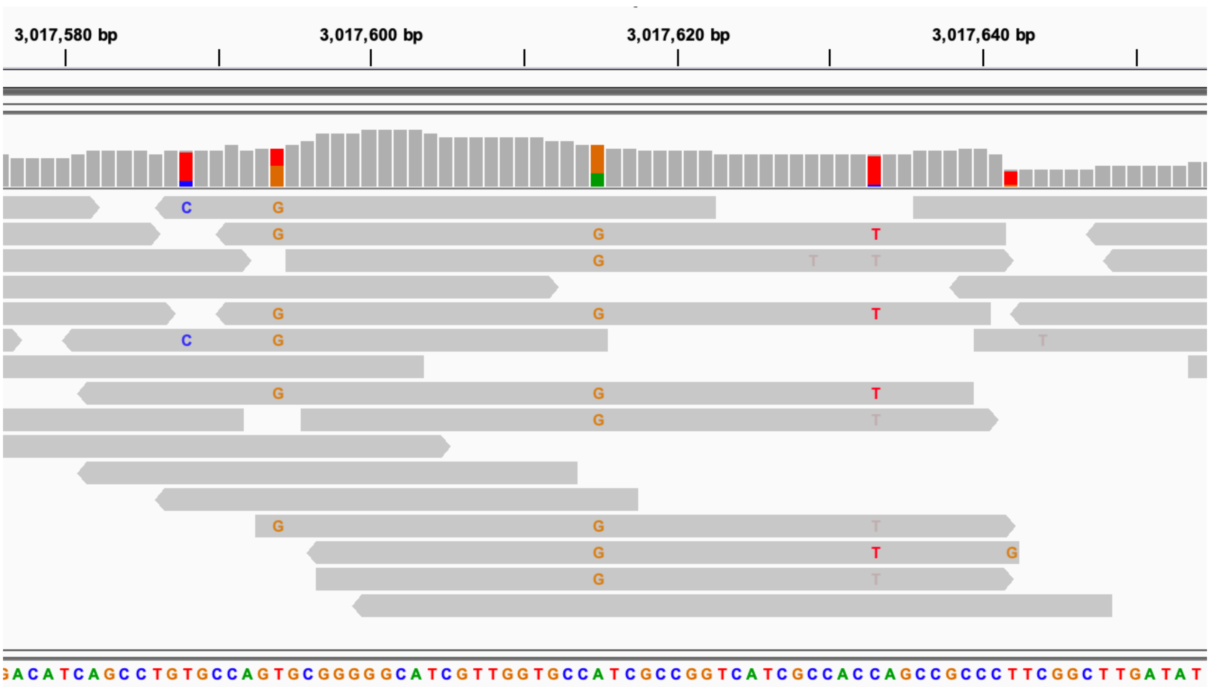

Supplementary Fig. 8 - False-positive private SNP call identified in BSK001/003. Visual inspection of position C3017633T identified in BSK001/003 was performed using IGV[119]. The variant was identified as unique, when compared against a set of 203 modern and 47 ancient *Y. pestis* genomes.

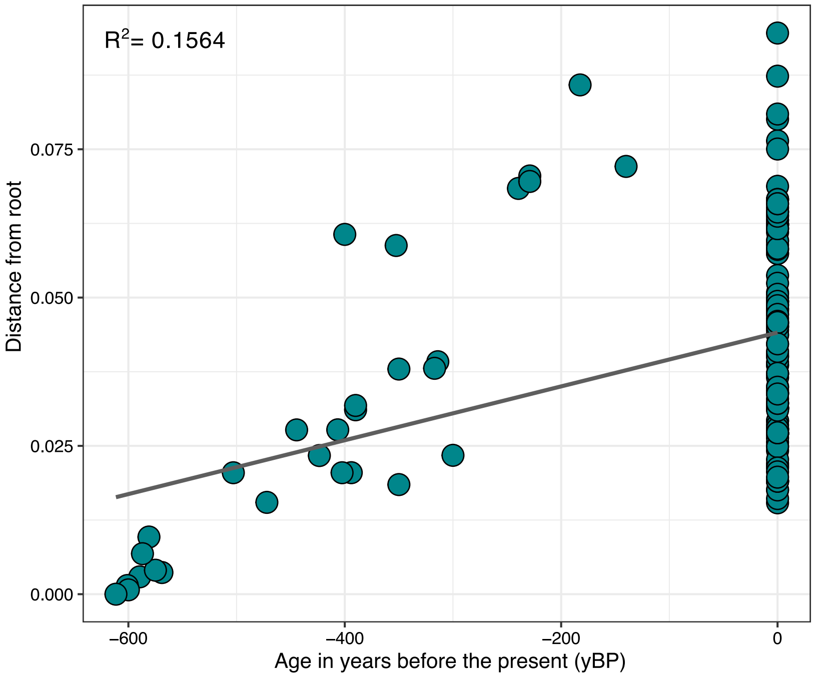

Supplementary Fig. 9 – Temporal signal regression analysis of root-to-tip distance against specimen age. All panels were created using the ggplot2[117] package on R version 3.6.1[118].

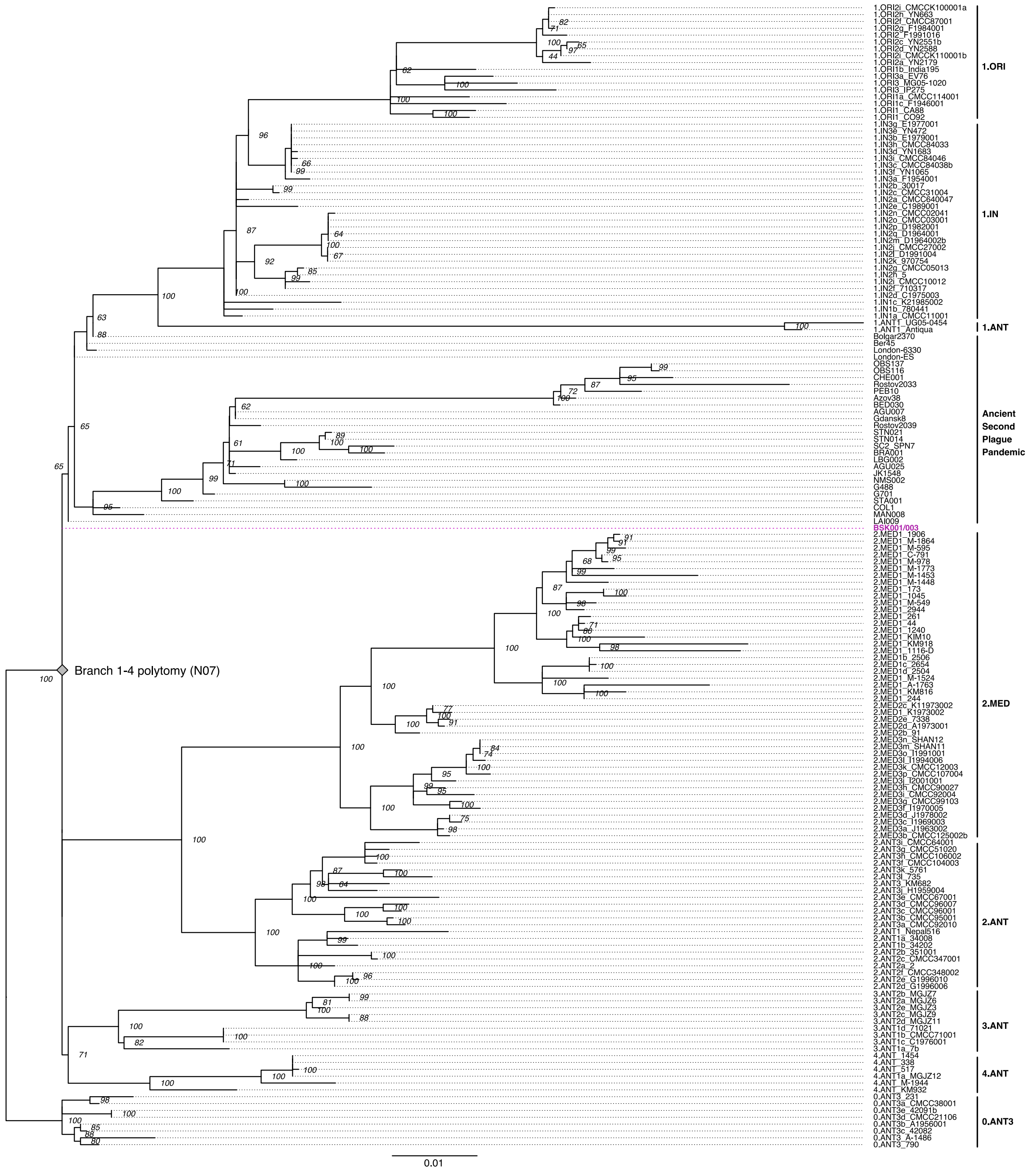

Supplementary Fig. 10 – *Y. pestis* dataset used for molecular dating analysis. Maximum likelihood phylogenetic tree constructed with RaxML[120] using 1,000 bootstrap iterations for estimating node support. The tree is constructed with 167 modern and historical *Y. pestis* genomes, and is based on 1,405 SNPs (95% partial deletion).

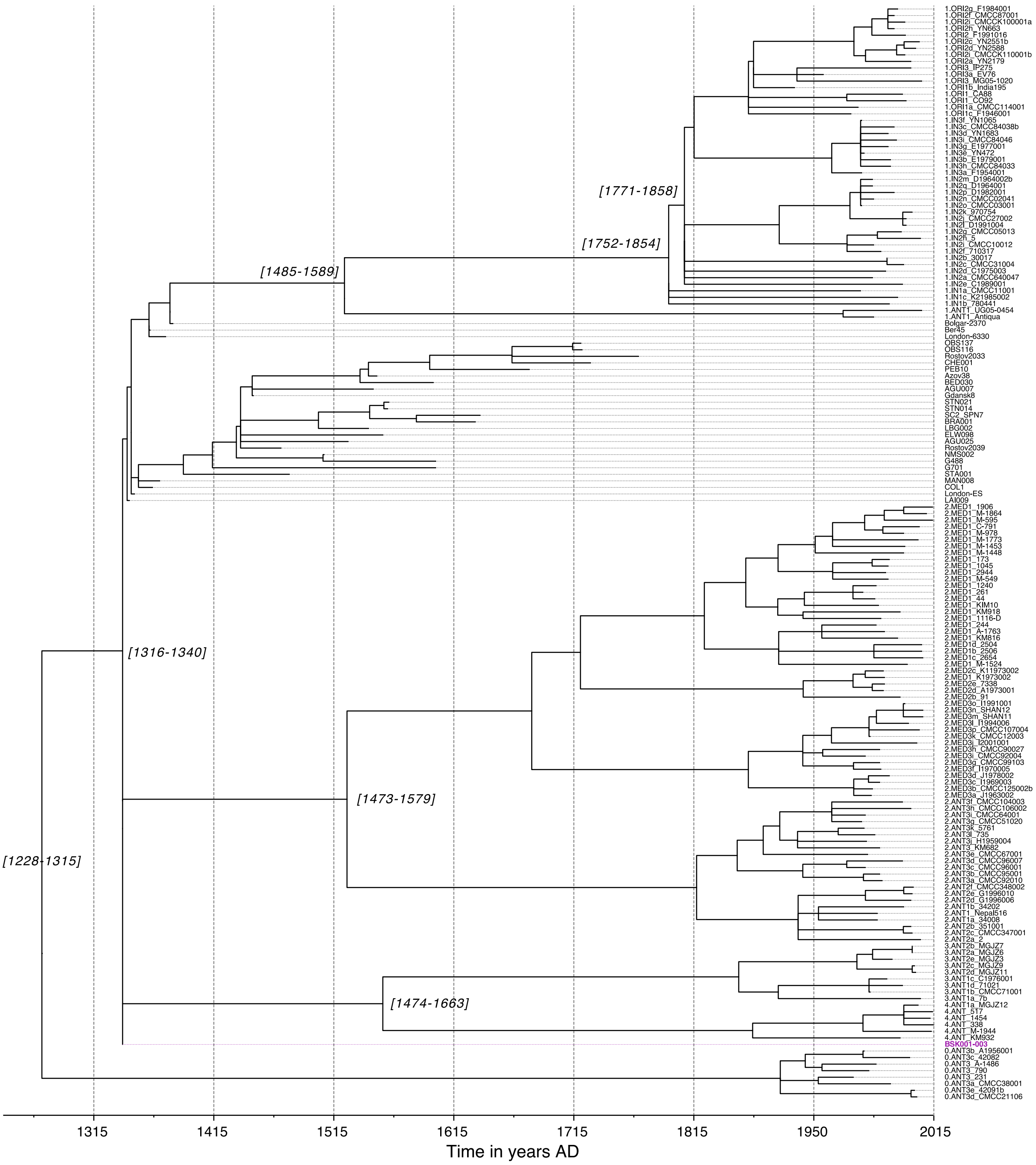

Supplementary Fig. 11 – Time-calibrated phylogeny computed with the program TreeTime[121]. The tree is based on 138 modern and 29 historical *Y. pestis* genomes. See Supplementary Table 22 for all used tip dates.

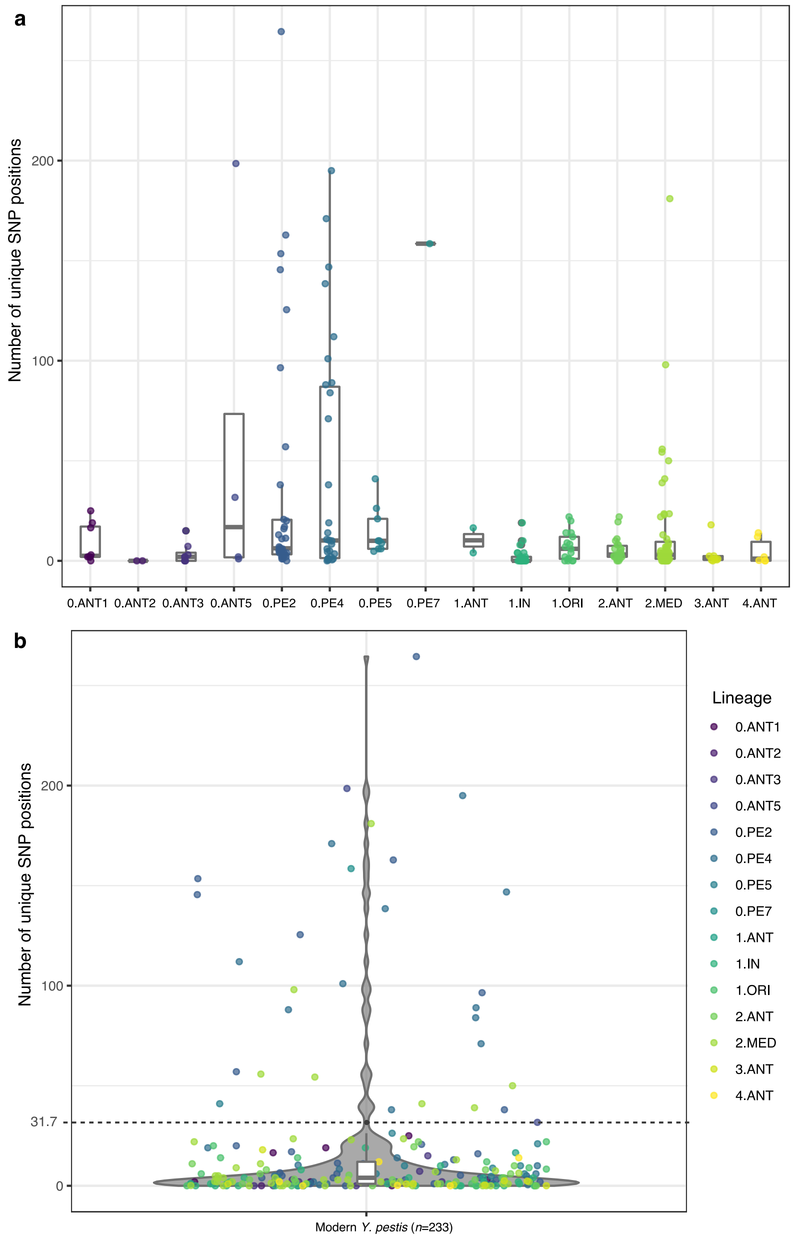

Supplementary Fig. 12 – Private branch length distribution across 233 modern *Y. pestis* genomes. The private branch lengths of *Y. pestis* genomes used as a comparative dataset in this study were used as a means for filtering out modern-genome assemblies with potential false-positive SNP calls (see Methods section *Phylogenetic reconstruction and diversity estimations*). The private branch lengths of 233 modern genomes (see[122] for dataset description) were retrieved from a maximum parsimony phylogenetic tree constructed in MEGA7[123] (based on 6,032 variant positions with 95% partial deletion). **a,** The box plots show the distribution of private branch lengths in strains comprising previously designated phylogenetic lineages. Centre lines represent median values, the lower and upper box edges represent the first and third data quartiles, respectively, and whisker extensions are defined within 1.5× the interquartile range. **b,** Violin plot showing the distribution of private branch lengths across all analysed strains. The dotted line indicates the minimum number of private variants across 30 genomes that were defined as outliers based on the depicted boxplot (beyond 1.5× interquartile range). All strains with private branch lengths of ≥31.7 SNPs were excluded from further analyses. Both panels were created using the ggplot2[117] package on R version 3.6.1[118].

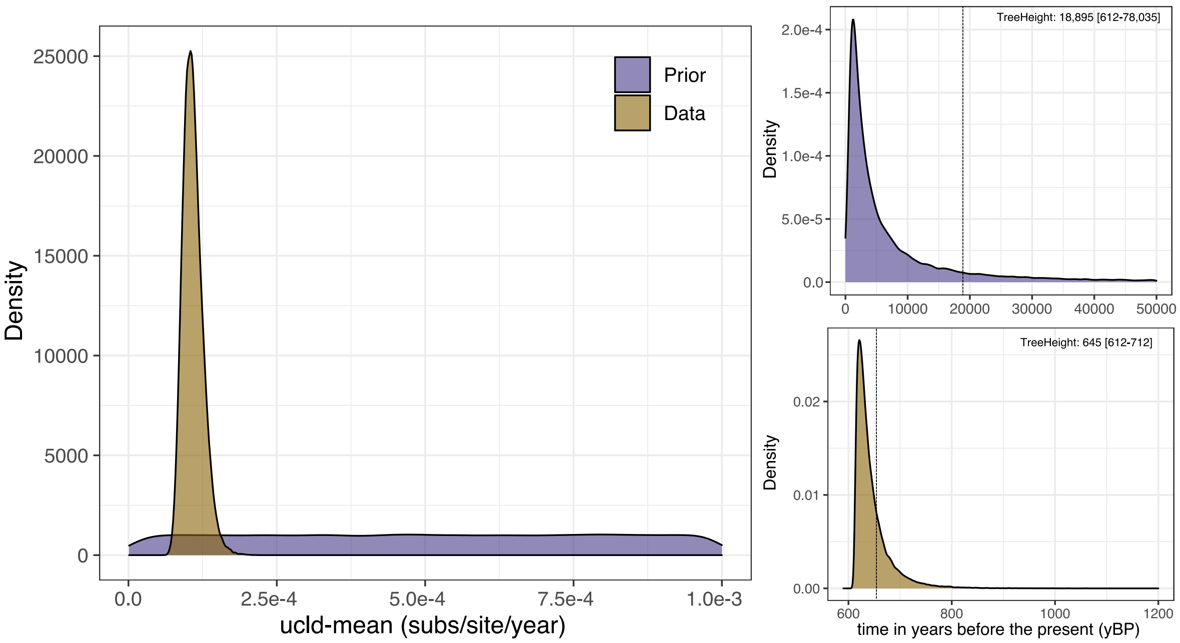

Supplementary Fig. 13 – Sampling from the prior analysis to assess robusticity of posterior estimates. The analysis was performed using BEAST2 v6.6. Posterior intervals are shown in brown for the data-informed analysis, whereas estimates after sampling from the prior are shown in purple. All panels were created using the forcats[124] and ggplot2[117] packages on R version 3.6.1[118].

Supplementary table descriptions

All supplementary tables of this study are provided in spreadsheet format.

Supplementary Table 1 – Annual distribution of dated tombstones identified in Kara-Djigach and Burana

Supplementary Table 2 – Mapping of shotgun-sequenced data against the human reference genome (*Hg19*)

Supplementary Table 3 – Mapping of sequenced data against the human reference genome (*Hs37d5*) after enrichment of 1.24 million SNP sites and estimates of relatedness

Supplementary Table 4 – Human ancestry modelling performed with the qpWave/qpAdm framework of grouped individuals BSK001, BSK003 and BSK005. P-values are obtained with chi-square tests on the matrix of f4-statistics.

Supplementary Table 5 – Individual-based human ancestry modelling performed with the qpWave/qpAdm framework for BSK001, BSK002, BSK003 and BSK005. P-values are obtained with chi-square tests on the matrix of f4-statistics.

Supplementary Table 6 – Taxonomic nodes targeted through HOPS, which show indications of reads with potential aDNA-associated damage and a declining edit distance.

Supplementary Table 7 – Summary of metagenomic read assignments to the *Y. pseudotuberculosis* complex and *Y. pestis* taxonomic nodes as determined by the pipeline HOPS

Supplementary Table 8 – Mapping of shotgun-sequenced data against the *Yersinia pestis* CO92 reference chromosome (NC_003143.1)

Supplementary Table 9 – Mapping of sequenced data against the *Y. pestis* chromosome (NC_003143.1) after whole-genome enrichment

Supplementary Table 10 – Mapping of sequenced data against the *Y. pestis* plasmids (pMT1 NC_003134.1, pPCP1 NC_003132.1 and pCD1 NC_003131.1) after enrichment

Supplementary Table 11 – Taxonomic classification of post-capture reads mapping against *Y. pestis* (node summarised reads in MEGAN)

Supplementary Table 12 – SNP calls in BSK003 differing from BSK001

Supplementary Table 13 – *Y. pestis* comparative genome dataset used for phylogenetic analysis in this study

Supplementary Table 14 – Table of variant positions in BSK001 and BSK003

Supplementary Table 15 – Plasmid variants across historical *Y. pestis* genomes

Supplementary Table 16 – Private SNPs in BSK001/003 prior to metagenomic (MALT) filtering

Supplementary Table 17 – Evaluation of private SNPs in BSK001/003 after metagenomic (MALT) filtering

Supplementary Table 18 – Phylogenetically diagnostic SNPs in ancient and modern *Y. pestis* genomes. SNPs were called at a minimum threshold of 3-fold. For Ns in BSK001, BSK003 and BSK001-003, the proportion of supporting reads for 1-fold, 2-fold and low-quality calls are shown in brackets.

Supplementary Table 19 – Age ranges of ancient *Y. pestis* genomes used for molecular dating analysis with BEAST2 v6.6

Supplementary Table 20 – Marginal likelihood estimates and log Bayes factor calculations for evaluation of all demographic models tested in BEAST2 v6.6

Supplementary Table 21 – 0.ANT genotyped strains and their isolation details

Supplementary Table 22 – Tip dates used as input for the time-calibrated phylogeny inferred by TreeTime

Supplementary References

1 De Guignes, J. *Histoire générale des Huns, des Turcs, des Mongols et des autres Tartares occidentaux*. (Desaint & Saillant, 1758).

2 Sprengel, K. *Beiträge zur Geschichte der Medicin*. Vol. 1 (In der Rengerschen Buchhandlung, 1794).

3 Schnurrer, F. *Chronik der Seuchen in Verbindung mit den gleichzeitigen Vorgängen*. (Christian Friedrich Osiander, 1823).

4 Hecker, J. F. C. *Der schwarze Tod im vierzehnten Jahrhundert: nach den Quellen für Aerzte und gebildete Nichtärzte*. (Herbig, 1832).

5 Gasquet, F. A. *The Great Pestilence (A.D. 1348-9), Now Commonly Known as the Black Death*. (Simpkin Marshall, 1893).

6 Nohl, J. *Der Schwarze Tod: eine Chronik der Pest 1348-1720, unter Benutzung zeitgenössischer Quellen bearbeitet*. (Gustav Kiepenheuer, 1924).

7 Langer, L. N. The Black Death in Russia: its effects upon urban labor. *Russian History* **2**, 53-67, doi:10.1163/187633175X00045 (1975).

8 Schamiloglu, U. in *Varia Eurasiatica: Festschrift für Professor András Róna-Tas* Ch. The End of Volga Bulgarian. , 157-164 (1991).

9 Schamiloglu, U. Preliminary Remarks on the Role of Disease in the History of the Golden Horde. *Central Asian Survey* **12**, 447-457 (1993).

10 Morelli, G. *et al.* Yersinia pestis genome sequencing identifies patterns of global phylogenetic diversity. *Nat Genet* **42**, 1140-1143 (2010).

11 Stewart, J. *Nestorian Missionary Enterprise: The story of a Church on fire*. 198–213 (Edinburgh: T. & T. Clark, 1928).

12 Vernadsky, G. *The Mongols and Russia* (Yale University Press, 1953).

13 Haeser, H. *Lehrbuch des Geschichte der Medicin unde der epidemischen Krankheiten, Dritter Band* (Verlag von Gustav Fischer, 1882).

14 Cui, Y. *et al.* Historical variations in mutation rate in an epidemic pathogen, Yersinia pestis. *Proceedings of the National Academy of Sciences* **110**, 577-582 (2013).

15 Hymes, R. Epilogue: a hypothesis on the East Asian beginnings of the Yersinia pestis polytomy. *The Medieval Globe* **1**, 12 (2014).

16 Campbell, B. M. *The great transition. Climate, Disease and Society in the Late-Medieval World*. (Cambridge: Cambridge University Press, 2016).

17 McNeill, W. *Plagues and Peoples* (Anchor Books, 1976).

18 Chwolson, D. Syrisch-nestorianische Grabinschriften aus Semirjetschie. *Mémoires de l'Académie Impériale des Sciences de St.-Pétersbourg. VII ser.* **XXXVII:8** (1890).

19 Chwolson, D. *Syrisch-nestorianische Grabinschriften aus Semirjetschie, neue Folge*. (Commissionnaires de l'Académie impériale des sciences, 1897).

20 Wu, L.-t. in *Far Eastern Association of Tropical Medicine, Transactions of the Fifth Biennial Congress Held at Singapore, 1923* (eds A. L. Hoops & J. W. Scharff) Ch. The Original Home of Plague, 286-304 (John Bale, Sons & Danielsson, 1924).

21 Wu, L.-t. *A Treatise on Pneumonic Plague*. (Publications of the League of Nations, 1926).

22 Kalina, G. P. *Chuma v Srednei Azii.*, p. 9-10 (Arkhangelsk, 1936).

23 Pollitzer, R. Plague studies: I. A summary of the history and a survey of the present distribution of the disease. *Bull WHO* **4**, 475 (1951).

24 Hirst, L. F. *The Conquest of Plague. A Study of the Evolution of Epidemiology* (At the Clarendon Press, 1953).

25 Carpentier, E. Autour de la peste noire: famines et épidémies dans l'histoire du XIVe siècle. *Annales. Economies, sociétés, civilisations* **17**, 1062-1092 (1962).

26 Ackerknecht, E. H. *History and Geography of the Most Important Diseases*. (Hafner, 1965).
27 Deaux, G. *The Black Death, 1347*. (Hamish Hamilton, 1969).
28 Biraben, J.-N. *Les hommes et la peste en France et dans les pays européens et méditerranéens*. (Mouton, 1975).
29 Aberth, J. *From the Brink of the Apocalypse. Confronting Famine, War, Plague, and Death in the Later Middle Ages* (Routledge, 2000).
30 Aberth, J. *Plagues in World History* (Rowman & Littlefield Publishers, 2011).
31 Schmid, B. V. *et al.* Climate-driven introduction of the Black Death and successive plague reintroductions into Europe. *Proc Natl Acad Sci U S A* **112**, 3020-3025 (2015).
32 Dols, M. W. *The Black Death in the Middle East* (Princeton University Press, 1977).
33 Horrox, R. *The Black Death*. (Manchester University Press, 1994).
34 Ziegler, P. *The Black Death*. (New York: John Day Company, 1969).
35 Kelly, J. *The Great Mortality. An Intimate History of the Black Death, the Most Devastating Plague of All Time* (Harper Collins, 2005).
36 Suntsov, V. V. & Suntsova, N. I. *Chuma. Proiskhozhdeniye i Evolutsiya Epizooticheskoi Sistemy* (Tovarishchestvo Nauchnykh Izdanii KMK, 2006).
37 Slavin, P. Death by the Lake: Mortality Crisis in Early Fourteenth-Century Central Asia. *Journal of Interdisciplinary History* **50**, 59-90 (2019).
38 Green, M. H. The Four Black Deaths. *The American Historical Review* **125**, 1601-1631 (2020).
39 Green, M. H. How a microbe becomes a pandemic: a new story of the Black Death. *The Lancet Microbe* **1**, e311-e312 (2020).
40 Fancy, N. & Green, M. H. Plague and the Fall of Baghdad (1258). *Medical history* **65**, 157-177 (2021).
41 Creighton, C. *History of Epidemics in Britain: From A.D. 664 to the Extinction of Plague by Creighton*. (Cambridge University Press, 1891).
42 Simpson, W. J. *A Treatise on Plague : Dealing with the Historical, Epidemiological, Clinical, Therapeutic and Preventive Aspects of the Disease*. (Cambridge University Press, 1905).
43 Mironov, N. P. The Past Existence of Natural Foci of Plague in the Steppes of Southern Europe. *Journal of Microbiology, Epidemiology, and Immunobiology* **29**, 1193-1198 (1958).
44 Fyodorov, V. N. The Question of the Existence of Natural Foci of Plague in Europe in the Past. *Journal of Hygiene, Epidemiology, Microbiology, and Immunology* **4**, 135-141 (1960).
45 Norris, J. East or west? The geographic origin of the Black Death. *Bulletin of the History of Medicine* **51**, 1-24 (1977).
46 Benedictow, O. J. *The Black Death, 1346-1353: The complete history*. (Woodbridge: Boydell & Brewer, 2004).
47 Benedictow, O. J. *The Complete History of the Black Death*. (Boydell Press, 2021).
48 Benedictow, O. J. Yersinia pestis, the bacterium of plague, arose in East Asia. Did it spread westwards via the Silk Roads, the Chinese maritime expeditions of Zheng He or over the vast Eurasian populations of sylvatic (wild) rodents? *Journal of Asian History* **47**, 1-31 (2013).
49 Benedictow, O. J. Plague in the Late Medieval Nordic Countries. Epidemiological Studies. *Oslo, Middlealderforlaget* (1992).
50 Alexander, J. T. *Bubonic plague in early modern Russia: public health and urban disaster*. (Oxford University Press, 1980).

51 Suponitskii, M. B. & Suponitskaya, N. S. *Ocherki Istorii Chumy*. (Vuzovskaya Kniga, 2006).
52 Khaydarov, T. F. in *Zolotoordynskoye Naslediye. Materialy Mezhdunarodnoi Nauchnoi Konferentsii "Transformatsiya Politiko-Etnicheskoi Karty Vostochnoi Yevropy: Velikaya Vengriya, Volzhskaya Bulgariya i Obrazovaniye Zolotoi Ordy": Sbornik Statei* (ed I. M. Mirgaleyev) Ch. Rol' Ordynsko-Russkoi Politii I Krupnykh Epidemicheskikh Vspyshek Chumy V Istorii Tatar, pp. 190-226 (Kazan: Institut Istorii im. Sh. Mardzhani, 2021).
53 Hirsch, A. *Handbuch der historisch-geographischen Pathologie*. (Verlag von Ferdinand Enke, 1859).
54 Shrewsbury, J. F. D. *A History of Bubonic Plague in the British Isles*. (Cambridge University Press, 1971).
55 Aboudharam, G. *et al.* in *Peste: entre épidémie et sociétés/Plague: Epidemics and Societies* (eds Michel Signoli *et al.*) 207–215 (Firenze University Press, 2007).
56 Sticker, G. *Abhandlungen aus der Seuchengeschichte. I. Band: Die Pest* (Alfred Töpelmann, 1909).
57 Namouchi, A. *et al.* Integrative approach using Yersinia pestis genomes to revisit the historical landscape of plague during the Medieval Period. *Proceedings of the National Academy of Sciences* **115**, 201812865, doi:10.1073/pnas.1812865115 (2018).
58 Gottfried, R. S. *The Black Death: Natural and Human Disaster in Medieval Europe*. (The Free Press, 1983).
59 Barker, H. Laying the Corpses to Rest: Grain, Embargoes, and Yersinia pestis in the Black Sea, 1346–48. *Speculum* **96**, 97-126 (2021).
60 Atwood, C. P. Validation by holiness or sovereignty: Religious toleration as political theology in the Mongol world empire of the thirteenth century. *The International History Review* **26**, 237-256 (2004).
61 Biran, M. *The Empire of the Qara Khitai in Eurasian History: Between China and the Islamic World*. (Cambridge University Press, 2005).
62 Akindinov, S. V., Kamyshev, A.M., and Petrov, P.N. Balasagun i Ordu - Monetnyye Dvory Vtoroi Chetverti XIII Veka. *Epigrafika Vostoka* 14-28 (2011).
63 Petrov, P. N., Kamyshev, A.M., Akindinov, S.V. Balasagun i Orda - Monetnyye Dvory Chuiskoi Doliny Pervoi Treti XIII Veka. *Izvestiya Natsional'noi Akademii Nauk Respubliki Kazakhstan* 168-177 (2012).
64 Chwolson, D. Syrische Grabinschriften aus Semirjetschie. *Memoires de l'Académie Impériale des Sciences de St. Petersbourg* **XXXIV:4**, pp. 1-30 (1886).
65 Jackson, P. *The Mission of Friar William of Rubruck*. p. 163 (London, 1990).
66 Yule, H. *The book of Ser Marco Polo, the Venetian, concerning the kingdoms and marvels of the East*. Vol. 2 284-5 (London, 1903).
67 Petrov, P. N., Kamyshev, A.M. in *Margulanovskiye Chteniya - 2020: Materialy Mezhdunarodnoi Nauchno-Prakticheskoi Konferentsii 'Velikaya Step' v Svete Arkheologicheskikh i Mezhdistsiplinarnykh Issledovanii* Vol. 1 (ed B.A. Baitanayev) Ch. Novyi Chagataidskii monetnyi dvor XIII-XIV vv. (k numizmatike gorodishcha Kara-Dzhigach), 266-285 (Almaty, 17-18 Sentyabrya., 2020).
68 Pantusov, N. Khristiyanskoe kladbishche bliz goroda Pishpeka (Semirechenskoi Oblasti) v Chuiskoi doline. *Zapiski Vostochnago Otdeleniya Imperatorskago Russkago Arkheologicheskago Obshchestva* **1**, pp. 74-83 (1886).
69 Fride, A. in *I.M. Samigulin* (ed 1857-1912) Ch. Ch. Predpisaniye Voyennogo Gubernatora Semirechenskoi Oblasti A. Ya. Fride Inspektoru Narodnykh Uchilishch D.V. Gorodetskomu o Zaprishchenii Vyvoza Nestorianskikh Nadgrobnykh

Pamiatnikov bez Razresheniia Vysshego Nachal'stva, pp. 150-151 (Almaty: LEM: 2011).
70 Fedotova, I. V., Dickens, M., Dickens, M., Avtushkova, A.L. in *Trudy VI (XXII) Vserossiiskogo Arkheologicheskogo S'yezda v Samare, Tom III* (ed N.A. Makarov A.P Derevianko, O.D. Mochalov) Ch. Kairaki v Sobranii Sibirskikh Muzeyev, pp. 61-63 (Samara: Minesterstvo Kul'tury Samarskoi Oblasti 2020).
71 Thacker, T. W. A Nestorian Gravestone from Central Asia in the Gulbenkian Museum, Durham University. *The Durham University Journal* **59** 94-107 (1967).
72 Ludevig, Y. *Spisok cherepam kraniologicheskoi kollektsii Muzeya Antropologii i Etnografii imeni Imperatora Petra Velikago*. (St Petersburg, 1904).
73 Slutskii, S. Semirechenskiya Nestoriyanskiya Nadpisi. *Drevnosti Vostochnyya* **1** pp. 1-72 (1889).
74 Debets, G. F. *Paleoantropologiya SSSR*. (Moscow, 1948).
75 Pantusov, N. in *Arkheologiya Semirech'ya, 1857-1912 gg.* (ed I.M. Samigulin) Ch. Otnosheniye Starshego Chinovnika Osobykh Poruchenii pri Voyennom Gubernatore Semirechenskoy Oblasti N.N. Pantusova v Imperatorskuyu Arkheologicheskuyu Kommissiyu o Khode Raskopok na Nestorianskom Kladbishche vblizi Pishpeka, pp. 191-192 (Almaty: LEM: 2011).
76 Klein, W. *Das nestorianische Christentum an den Handelswegen durch Kyrgyzstan bis zum 14. Jh.*, (Turnhout, 2000).
77 Pantusov, N. in *Arkheologiya Semirech'ya, 1857-1912* (ed I.M. Samigulin) Ch. Otchet o Rabotakh po Issledovaniyu Pripishpekskogo Nestorianskogo Kladbishcha v Sentyabre-Oktyabre 1892 g., pp. 349-351 (Almaty: LEM: 2011).
78 Chaffanjon, J. Rapport sur une mission scientifique dans l'Asie centrale et la Sibérie. *Nouvelles archives des missions scientifiques et littéraires* **9**, pp. 55-101 (1899).
79 Nau, F. Les pierres tombales nestoriennes du Musée Guimet. *Revue de l'Orient Chrétien* **8**, 3-35 (1913).
80 Hjelt, A. Drei Syrisch-Nestorianische Grab-Inschriften. *Annales Academiae Scientiarum Fennicae S. B* **1**, 3-11 (1909).
81 Kokovtsov, P. K. K Siro-Turetskoi Epigrafike Semirech'ya. *Izvestiya Imperatorskoi Akademii Nauk* **3**, 773-796 (1909).
82 Dzhumagulov, C. *Epigrafika Kirgizii, Vypusk 3*. (Frunze, 1987).
83 Dzhumagulov, C. *Yazyk Siro-Tyurkskikh (Nestorianskikh) Pamyatnikov Kirgizii* (Frunze, 1971).
84 Dzhumagulov, C. *Epigrafika Kirgizii, Vypusk 2*. (Frunze, 1982).
85 Dzhumagulov, C. *Kyrgyzstandagy nestorian-turk zhazuu estelikteri (XIII-XIV kylymdar)*. (Bishkek, 2011).
86 Dickens, M. (ed D. Winkler and L. Tang) Ch. Syriac Gravestones in the Tashkent Museum, 13-49 (Münster, 2009).
87 Borbone, P. G. Three Notes on Syro-Turkic Epigraphy. *Khristiyanskii Vostok* **9**, 105-122 (2021).
88 Dzhumagulov, C. Die syrisch-türkischen (nestorianischen) Denkmäler in Kirgisien. *Mitteilungen des Instituts für Orientforschung* **14**, 470–480 (1968).
89 Fedorov, M. A Hoard of Fourteenth Century Chaghatayid Silver coins from North Kirghizstan. *The Numismatic Chronicle* **162**, 404-419 (2002).
90 Ochkasov, Y. S. Komplekt monet Chagataidov XIII-XIV vv. iz Chuiskoi doliny. *Numizmatika Zolotoi Ordy* **6**, pp. 109-112 (2016).
91 Yadrintsev, N. Arkheologicheskiya izyskaniya i otkrytiya d-ra Poyarkova bliz Tokmaka. *Zapiski Imperatorkoi Akademii Nauk* **52**, pp. 152-164 (1886).

92 Marr, N. Nadgrobnyi kamen' iz Semirechiya s Armyanskoi-Siriiskoi Nadpis'yu 1323 g. *Zapiski Vostochnago Otdeleniya Imperatorskago Russkago Arkheologicheskago Obshchestva* **8**, 344-349 (1894).
93 Peltzer, A. *et al.* EAGER: efficient ancient genome reconstruction. *Genome biology* **17**, 60 (2016).
94 Schubert, M., Lindgreen, S. & Orlando, L. AdapterRemoval v2: rapid adapter trimming, identification, and read merging. *BMC research notes* **9**, 88 (2016).
95 Li, H. & Durbin, R. Fast and accurate short read alignment with Burrows–Wheeler transform. *Bioinformatics* **25**, 1754-1760 (2009).
96 Li, H. *et al.* The sequence alignment/map format and SAMtools. *Bioinformatics* **25**, 2078-2079 (2009).
97 Skoglund, P., Storå, J., Götherström, A. & Jakobsson, M. Accurate sex identification of ancient human remains using DNA shotgun sequencing. *Journal of Archaeological Science* **40**, 4477-4482 (2013).
98 Jónsson, H., Ginolhac, A., Schubert, M., Johnson, P. L. & Orlando, L. mapDamage2. 0: fast approximate Bayesian estimates of ancient DNA damage parameters. *Bioinformatics* **29**, 1682-1684 (2013).
99 Korneliussen, T. S., Albrechtsen, A. & Nielsen, R. ANGSD: analysis of next generation sequencing data. *BMC Bioinformatics* **15**, 356 (2014).
100 Renaud, G., Slon, V., Duggan, A. T. & Kelso, J. Schmutzi: estimation of contamination and endogenous mitochondrial consensus calling for ancient DNA. *Genome biology* **16**, 1-18 (2015).
101 Kloss-Brandstätter, A. *et al.* HaploGrep: a fast and reliable algorithm for automatic classification of mitochondrial DNA haplogroups. *Hum Mutat* **32**, 25-32 (2011).
102 Jun, G., Wing, M. K., Abecasis, G. R. & Kang, H. M. An efficient and scalable analysis framework for variant extraction and refinement from population-scale DNA sequence data. *Genome Res* **25**, 918-925 (2015).
103 Patterson, N. *et al.* Ancient admixture in human history. *Genetics* **192**, 1065-1093 (2012).
104 Lazaridis, I. *et al.* Genomic insights into the origin of farming in the ancient Near East. *Nature* **536**, 419-424 (2016).
105 Jeong, C. *et al.* The genetic history of admixture across inner Eurasia. *Nature ecology & evolution* **3**, 966-976 (2019).
106 Patterson, N., Price, A. L. & Reich, D. Population structure and eigenanalysis. *PLoS Genet* **2**, e190 (2006).
107 Kennett, D. J. *et al.* Archaeogenomic evidence reveals prehistoric matrilineal dynasty. *Nature communications* **8**, 1-9 (2017).
108 Kuhn, M., Manuel, J., Jakobsson, M. & Günther, T. Estimating genetic kin relationships in prehistoric populations. *PloS one* **13**, e0195491 (2018).
109 Haak, W. *et al.* Massive migration from the steppe was a source for Indo-European languages in Europe. *Nature* **522**, 207-211 (2015).
110 Reich, D. *et al.* Reconstructing native American population history. *Nature* **488**, 370-374 (2012).
111 Quintana-Murci, L. *et al.* Where west meets east: the complex mtDNA landscape of the southwest and Central Asian corridor. *The American Journal of Human Genetics* **74**, 827-845 (2004).
112 Derenko, M. *et al.* Complete mitochondrial DNA diversity in Iranians. *PloS one* **8**, e80673 (2013).
113 de Barros Damgaard, P. *et al.* 137 ancient human genomes from across the Eurasian steppes. *Nature* **557**, 369 (2018).

114 Gnecchi-Ruscone, G. A. *et al.* Ancient genomic time transect from the Central Asian Steppe unravels the history of the Scythians. *Science Advances* **7**, eabe4414 (2021).
115 Ning, C. *et al.* Ancient genomes from northern China suggest links between subsistence changes and human migration. *Nature communications* **11**, 1-9 (2020).
116 Jeong, C. *et al.* A dynamic 6,000-year genetic history of Eurasia's Eastern Steppe. *Cell* **183**, 890-904. e829 (2020).
117 Wickham, H. *ggplot2: elegant graphics for data analysis*. (Springer, 2016).
118 RCoreTeam. R: A language and environment for statistical computing. *R Foundation for Statistical Computing, Vienna, Austria.*, doi:https://www.R-project.org/ (2019).
119 Thorvaldsdóttir, H., Robinson, J. T. & Mesirov, J. P. Integrative Genomics Viewer (IGV): high-performance genomics data visualization and exploration. *Briefings in bioinformatics* **14**, 178-192 (2013).
120 Stamatakis, A. RAxML version 8: a tool for phylogenetic analysis and post-analysis of large phylogenies. *Bioinformatics* **30**, 1312-1313 (2014).
121 Sagulenko, P., Puller, V. & Neher, R. A. TreeTime: Maximum-likelihood phylodynamic analysis. *Virus evolution* **4**, vex042 (2018).
122 Spyrou, M. A. *et al.* Phylogeography of the second plague pandemic revealed through analysis of historical Yersinia pestis genomes. *Nature communications* **10**, 1-13 (2019).
123 Kumar, S., Stecher, G. & Tamura, K. MEGA7: Molecular Evolutionary Genetics Analysis Version 7.0 for Bigger Datasets. *Mol Biol Evol* **33**, 1870-1874 (2016).
124 Wickham, H. Forcats: Tools for working with categorical variables (factors). *R package version 0.4. 0* (2019).
