# Supplementary material for: The source of the Black Death in fourteenth-century central Eurasia
Source: Nature. 2022 Jun 15;606(7915):718–24. doi: 10.1038/s41586-022-04800-3 (PMC9217749; doi:10.1038/s41586-022-04800-3)
Supplement: Supplementary file 2 — Reporting Summary [file 41586_2022_4800_MOESM2_ESM.pdf]

nature portfolio

Corresponding author(s): Maria A. Spyrou, Philip Slavin and Johannes Krause

Last updated by author(s): Mar 15, 2022

Reporting Summary

Nature Portfolio wishes to improve the reproducibility of the work that we publish. This form provides structure for consistency and transparency in reporting. For further information on Nature Portfolio policies, see our Editorial Policies and the Editorial Policy Checklist.

Statistics

For all statistical analyses, confirm that the following items are present in the figure legend, table legend, main text, or Methods section.

n/a	Confirmed	
☐	☒	The exact sample size (*n*) for each experimental group/condition, given as a discrete number and unit of measurement
☐	☒	A statement on whether measurements were taken from distinct samples or whether the same sample was measured repeatedly
☐	☒	The statistical test(s) used AND whether they are one- or two-sided<br>*Only common tests should be described solely by name; describe more complex techniques in the Methods section.*
☒	☐	A description of all covariates tested
☒	☐	A description of any assumptions or corrections, such as tests of normality and adjustment for multiple comparisons
☐	☒	A full description of the statistical parameters including central tendency (e.g. means) or other basic estimates (e.g. regression coefficient) AND variation (e.g. standard deviation) or associated estimates of uncertainty (e.g. confidence intervals)
☐	☒	For null hypothesis testing, the test statistic (e.g. *F*, *t*, *r*) with confidence intervals, effect sizes, degrees of freedom and *P* value noted<br>*Give P values as exact values whenever suitable.*
☐	☒	For Bayesian analysis, information on the choice of priors and Markov chain Monte Carlo settings
☒	☐	For hierarchical and complex designs, identification of the appropriate level for tests and full reporting of outcomes
☒	☐	Estimates of effect sizes (e.g. Cohen's *d*, Pearson's *r*), indicating how they were calculated

Our web collection on statistics for biologists contains articles on many of the points above.

Software and code

Policy information about availability of computer code

Data collection	No software was used for data collection
Data analysis	The following software was used for the data analysis portion of this study:<br>bcl2fastq v2.20, EAGER pipeline (version 1.92.58), FastQC version 0.11.4, AdapterRemoval v2.2.0, HOPS pipeline, MALT (v040), MEGAN (v6.4.12), BLASTn (web version), BWA (version 0.7.12), mapDamagev2.0, SAMtools v1.3, Picard version 1.140 MarkDuplicates, DeDup v0.12.2, QualiMap v.2.2.1, BBtools suite (https://sourceforge.net/projects/bbmap/), GATK v3.5, MultiVCFAnalyzer v0.85 (https://github.com/alexherbig/MultiVCFAnalyzer), R version 3.6.1, SNPEvaluation (build date 2018-08-13, https://github.com/andreasKroepelin/SNP_Evaluation), MEGA7, RAxML (version 8.2.9), TempEst v1.5.3, BEAST2 v6.6, Tracer v1.6, TreeTime v0.8.4, FigTree v1.4.4, GrapeTree version 1.5.0, Schmutzi (https://github.com/grenaud/schmutzi), HaploGrep2, pileupCaller v1.4.0 (https://github.com/stschiff/sequenceTools), bamUtil v.1.0.13, READ (https://bitbucket.org/tguenther/read/src/master/), qpWave/qpAdm (v1520), pMMR (https://github.com/TCLamnidis/pMMRCalculator), smartpca v16000, ANGSD v0.910, EIGENSOFT v6.0.1 and QGIS 3.22.1.<br>All listed software used for the data analysis portion of this study is publicly available.

For manuscripts utilizing custom algorithms or software that are central to the research but not yet described in published literature, software must be made available to editors and reviewers. We strongly encourage code deposition in a community repository (e.g. GitHub). See the Nature Portfolio guidelines for submitting code & software for further information.

Data

Policy information about availability of data

All manuscripts must include a data availability statement. This statement should provide the following information, where applicable:

- Accession codes, unique identifiers, or web links for publicly available datasets
- A description of any restrictions on data availability
- For clinical datasets or third party data, please ensure that the statement adheres to our policy

The raw sequence data produced in this study, the Y. pestis aligned reads after metagenomic filtering and the human aligned reads are available through the European Nucleotide Archive under accession number PRJEB46734. Additional data is available within the Supplementary Information section of this study. Comparative data including Y. pestis genome accessions can be found in Supplementary table 13 . Comparative human genomic data was retrieved from version v50.0 of the Allen Ancient DNA resource (https://reich.hms.harvard.edu/allen-ancient-dna-resource-aadr-downloadable-genotypes-present-day-and-ancient-dna-data).

Field-specific reporting

Please select the one below that is the best fit for your research. If you are not sure, read the appropriate sections before making your selection.

☒ Life sciences ☐ Behavioural & social sciences ☐ Ecological, evolutionary & environmental sciences

For a reference copy of the document with all sections, see nature.com/documents/nr-reporting-summary-flat.pdf

Life sciences study design

All studies must disclose on these points even when the disclosure is negative.

Sample size	Specimen sample size was determined on the basis of available skeletal material stored within the Kunstkamera, Peter the Great Museum of Anthropology and Ethnography, Russian Academy of Sciences, in St. Petersburg, Russia. The seven tooth specimens analysed in this study were evaluated on the basis of ancient DNA (aDNA) preservation using previously defined cirteria. Specimens with sufficient levels of aDNA preservation were used for whole-genome or genome-wide variant analysis. A detailed description of specimen analysis is provided within the Methods section of this study.
Data exclusions	Data from specimens that showed insufficient levels of ancient DNA preservation were excluded from further genomic analyses. Genomic analyses of specimens that showed sufficient ancient DNA preservation were carried out after read filtering according to previously defined ancient DNA criteria. In brief, raw sequenced reads that displayed poor sequencing quality and those shorter than 30 base pairs in length were excluded. In addition, reads of with low mapping quality (<30 for human mapping reads and <37 for Y. pestis mapping reads) were also filtered. Moreover, for the analysis of the newly generated Y. pestis genomes, a taxonomy-informed read filtering was implemented in this study in order to exclude DNA fragments that potentially stem from environmental microbial contamination. For the comparative dataset, present-day genomes showing possible presence of contaminant SNPs were excluded on the basis of their terminal branch lengths, whereby genome assemblies with excessively long branches were not considered for evolutionary analysis as their associated raw data could not be evaluated. A detailed description of data filtering is provided within the Methods section of this study.
Replication	For individuals where ancient Y. pestis DNA was detected, three genetic libraries were produced from each of the specimens BSK001 and BSK003, and one genetic library was generated from BSK007, all confirming the pathogen's presence. Evolutionary inferences were performed using different methods, including the maximum likelihood and Bayesian phylogenetic methods. Phylogenetic analyses were furthermore repeated using different comparative datasets. All methods used support the conclusions reported in this study. A detailed description is provided within the Methods section of this study.
Randomization	No experiment or analysis requiring allocation of samples/organisms or participants in random groups was carried out for this study.
Blinding	Blinding is not relevant to this study. No experiment or analysis requiring group allocations was carried out for this study.

Reporting for specific materials, systems and methods

We require information from authors about some types of materials, experimental systems and methods used in many studies. Here, indicate whether each material, system or method listed is relevant to your study. If you are not sure if a list item applies to your research, read the appropriate section before selecting a response.

Materials & experimental systems

n/a	Involved in the study	
☒	☐	Antibodies
☒	☐	Eukaryotic cell lines
☐	☒	Palaeontology and archaeology
☒	☐	Animals and other organisms
☒	☐	Human research participants
☒	☐	Clinical data
☒	☐	Dual use research of concern

Methods

n/a	Involved in the study	
☒	☐	ChIP-seq
☒	☐	Flow cytometry
☒	☐	MRI-based neuroimaging

Palaeontology and Archaeology

Specimen provenance

Excavations of the Kara Djigach and Burana cemeteries took place during the years 1885 and 1886 by N. Pantusov and A. Fetisov. Human skeletal remains have since the year 1937 been stored at Kunstkamera, Peter the Great Museum of Anthropology and Ethnography, Russian Academy of Sciences, in St. Petersburg, Russia. A detailed description of the excavations and sample provenance is included within the Supplementary Information section of this study. Samples from Kara Djigach (archaeological IDs: 176/4; 176/5; 176/7; 5559/1; 5559/2 , aDNA Jena lab IDs: BSK001, BSK002, BSK003, BSK006, BSK007) and from Burana (archaeological IDs: 188/1; 188/2, aDNA Jena lab IDs: BSK004, BSK005) were analysed within the ancient DNA clean room facilities of the Max Planck Institute for the Science of Human History,in Jena, Germany, with permission from Kunstkamera, Peter the Great Museum of Anthropology and Ethnography, Russian Academy of Sciences.

Specimen deposition

The skeletal assemblages associated with the Kara Djigach and Burana archaeological sites are kept within the collection of the Kunstkamera, Peter the Great Museum of Anthropology and Ethnography.

Dating methods

Human skeletal remains have been precisely dated on the basis of associated burial tombstones. Detailed translations of all tombstone inscriptions associated with the analysed burials are provided within the Supplementary Information section of this study. Moreover, radiocarbon dating was performed for individuals BSK003 and BSK007 that were positive for Y. pestis. Radiocarbon dating was performed in the Curt-Engelhorn-Zentrum Archäometrie gGmbH in Mannheim, Germany. Collagen was extracted from the tooth roots (modified Longin method) and purified by ultrafiltration (fraction >30kD). Resulting dates were calibrated using the dataset IntCal20 and the software SwissCal (L.Wacker, ETH-Zürich). The corresponding laboratory IDs, uncalibrated radiocarbon dates and 2-sigma (95.45%) probability intervals are provided in Supplementary Information 2 of this study.

☒ Tick this box to confirm that the raw and calibrated dates are available in the paper or in Supplementary Information.

Ethics oversight

Seven tooth specimens from the archaeological sites of Kara-Djigach (n=5) and Burana (n=2) have been analysed in the present study. Approvals for ancient DNA analysis have been obtained from the relevant custodians within the Kunstkamera, Peter the Great Museum of Anthropology and Ethnography, Russian Academy of Sciences, in St. Petersburg, who are co-authors in this paper and have approved the study protocol.

Note that full information on the approval of the study protocol must also be provided in the manuscript.
